# Metabolomics signatures of sweetened beverages and added sugar are related to anthropometric measures of adiposity in young individuals: results from a cohort study

**DOI:** 10.1016/j.ajcnut.2024.07.021

**Published:** 2024-07-24

**Authors:** Samuel Muli, Maike E Schnermann, Mira Merdas, Jodi Rattner, David Achaintre, Ines Perrar, Jantje Goerdten, Ute Alexy, Augustin Scalbert, Matthias Schmid, Anna Floegel, Pekka Keski-Rahkonen, Kolade Oluwagbemigun, Ute Nöthlings

**Affiliations:** 1 Unit of Nutritional Epidemiology, Department of Nutrition and Food Sciences, University of Bonn, Bonn, Germany; 2 International Agency for Research on Cancer (IARC), Lyon, France; 3 Department of Epidemiological Methods and Etiological Research, Leibniz Institute for Prevention Research and Epidemiology (BIPS), Bremen, Germany; 4 Institute for Medical Biometry, Informatics and Epidemiology (IMBIE), University Hospital Bonn, Bonn, Germany; 5 Section of Dietetics, Faculty of Agriculture and Food Sciences, Hochschule Neubrandenburg, Neubrandenburg, Germany

**Keywords:** metabolite biomarkers, metabolomics, sweetened beverages, added sugar, adiposity

## Abstract

**Background:**

The associations of sweetened beverages (SBs) and added sugar (AS) intake with adiposity are still debated. Metabolomics could provide insights into the mechanisms linking their intake to adiposity.

**Objectives:**

We aimed to identify metabolomics biomarkers of intake of low- and no-calorie sweetened beverages (LNCSBs), sugar-sweetened beverages (SSBs), and ASs and to investigate their associations with body mass index, body fat percentage, and waist circumference.

**Methods:**

We analyzed 3 data sets from the Dortmund Nutritional and Anthropometric Longitudinally Designed (DONALD) cohort study, of children who provided 2 urine samples (*n* = 297), adolescents who provided a single urine sample (*n* = 339), and young adults who provided a single plasma sample (*n* = 195). Urine and plasma were analyzed using untargeted metabolomics. Dietary intakes were assessed using 3-d weighed dietary records. The random forest, partial least squares, and least absolute shrinkage and selection operator were jointly used for metabolite selection. We examined associations of intakes with metabolites and anthropometric measures using linear and mixed-effects regression.

**Results:**

In adolescents, LNCSB were positively associated with acesulfame (β: 0.0012; 95% confidence interval [CI]: 0.0006, 0.0019) and saccharin (β: 0.0009; 95% CI: 0.0002, 0.0015). In children, the association was observed with saccharin (β: 0.0016; 95% CI: 0.0005, 0.0027). In urine and plasma, SSBs were positively associated with 1-methylxanthine (β: 0.0005; 95% CI: 0.0003, 0.0008; and β: 0.0010, 95% CI 0.0004, 0.0015, respectively) and 5-acetylamino-6-amino-3-methyluracil (β: 0.0005; 95% CI: 0.0002, 0.0008; and β: 0.0009; 95% CI: 0.0003, 0.0014, respectively). AS was associated with urinary sucrose (β: 0.0095; 95% CI: 0.0069, 0.0121) in adolescents. Some of the food-related metabolomics profiles were also associated with adiposity measures.

**Conclusions:**

We identified SBs- and AS-related metabolites, which may be important for understanding the interplay between these intakes and adiposity in young individuals.

## Introduction

High consumption of sweetened beverages (SBs) or “soft drinks” and added sugars (ASs), particularly among children and adolescents has emerged as an important nutrition and public health issue [1]. SBs are generally divided into 2 categories, sugar-sweetened beverages (SSBs) and low-calorie and no-calorie sweetened beverages (LNCSBs). SSBs are a major source of ASs in the diet [2] and are argued to contribute to excess caloric intake and poor nutrition [3,4]. Regular consumption of SBs is associated with various health conditions, such as weight gain, obesity, type 2 diabetes, cardiovascular diseases, and some cancers [[5], [6], [7]]. However, some of these associations are not consistent across studies [8,9].

Dietary intake is typically assessed by self-reported dietary questionnaires, which are fraught with measurement errors [10]. Indeed, underreporting of SB and AS intake because of social desirability bias has been described [11]. In recent years, biomarkers of dietary intake have been proposed as one of the ways to improve dietary exposure assessment [12]. To date, however, few reliable biomarkers have been identified and validated for use in epidemiologic studies [[13], [14], [15]]. Some gains include the 24-hour urinary sucrose and fructose, which has been applied in calibrating total sugar intakes in diverse populations [16,17] although it does not distinguish intrinsic from AS. In our previous work [18], we outlined that the ^13^C:^12^C carbon isotope ratio (δ^13^C), measured in whole blood, red blood cells, hair, breath, and plasma correlates with AS and SSB intake. However, sucrose from C4 photosynthetic plants (e.g., corn, sugarcane) moderately correlates with δ^13^C compared with sucrose from C3 plants (e.g., sugar beets, most fruits). Thus, the utility of this biomarker is limited by the source of the sucrose [19]. Consistent with the earlier reviews [18,19], an updated review on validity of biomarkers of food intake emphasizes the sustained interest in discovery and validation of new biomarkers, particularly for foods like SSBs [20].

One issue that may have influenced the progress of biomarkers of dietary intake such as for SBs and AS is that many candidates are selected based on putative mechanisms. However, given the aforementioned potentially complex metabolism of these foods, targeting single or multiple selected pathways may be suboptimal. Large-scale metabolite measurement through untargeted metabolomics approaches across multiple data sets and biosamples could help uncover biomarkers of SBs and AS. Further, because metabolites of these foods might exist in a continuum in body fluids, profiling of the plasma and subsequently the urine could be an important research advance. Besides, changes in the metabolome are likely to represent important drivers of the relationship between the intake of intake of SBs and AS and adiposity. Interestingly, limited studies have investigated untargeted metabolomics biomarkers of SSB intake [[21], [22], [23], [24]], as well as the metabolic changes of SSB intake with adiposity [24].

Leveraging 3 data sets across 2 biosamples within a well-characterized cohort of children and adolescents, we aimed to explore metabolomics biomarkers of SBs and AS intake and to investigate their associations with 3 anthropometric measures of adiposity: BMI, body fat percentage (%BF), and waist circumference (WC).

## Methods

### Study design

The Dortmund Nutritional and Anthropometric Longitudinally Designed (DONALD) study is an open cohort in Dortmund, Germany, that has been recruiting infants in their first year of life since 1985. Participants undergo their first examination at 3 mo of age, followed by 3 additional visits in their first year of life, 2 visits in the second year, and then annually until young adulthood. Regular examinations include dietary intake, anthropometrics, urine samples (starting at age 3–4 y), blood samples (starting at age 18 y), and interviews on lifestyle, sociodemographics, and medical history. A more detailed description of the DONALD study is described elsewhere [25]. The DONALD study was approved by the Ethics Committee of the University of Bonn and conducted according to the guidelines of the Declaration of Helsinki. Written informed consent was obtained from the parents and from adolescents aged 16 y and above.

### Study population

**FIGURE 1:**
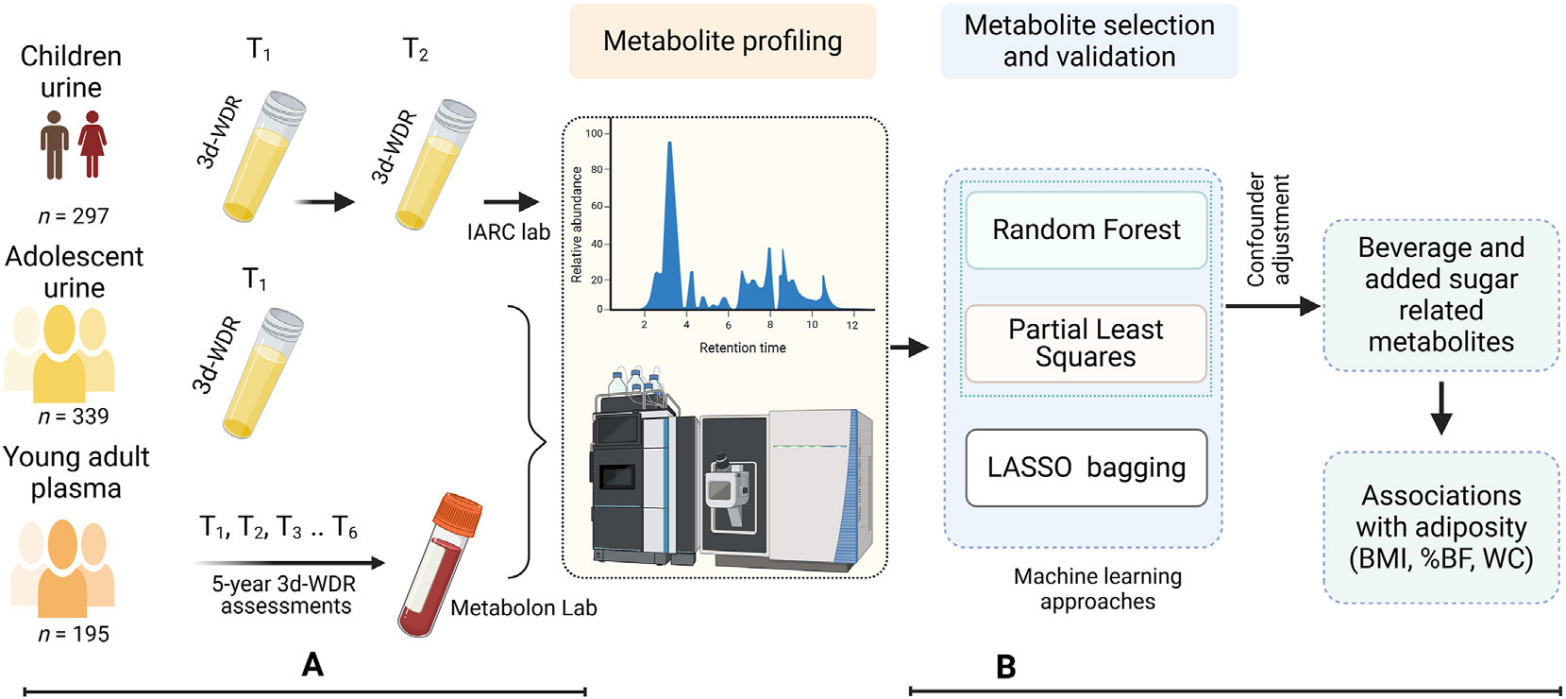
Study design and analysis. (A) Analytic samples and data collection. Children urine included 2 dietary assessments and 2 urine collections. Adolescent urine included single dietary assessment and urine collection. Young adult plasma included multiple (3–6) dietary assessments within 5 y preceding a single blood draw. (B) Study analytic plan. %BF, body fat percentage; 3d-WDR, 3-d weighed dietary record; LASSO, least absolute shrinkage and selection operator; T_1_–T_6_, dietary assessments; WC, waist circumference.

This analysis included 3 study samples, hereinafter termed children urine, adolescent urine, and young adult plasma. The eligible participants for children urine were individuals with two 3-d weighed dietary records (3d-WDRs) and 2 urine collections, and for adolescent urine, individuals with one 3d-WDR and 1 urine collection. Young adult plasma comprised individuals with 3 or more 3d-WDR assessments within the last 5 years preceding the date of blood draw. Figure 1 provides an overview of the 3 study samples and the analytical plan. Supplemental Figure 1 provides a detailed flowchart and the overlap of participants across the samples.

### Measures

#### Dietary intake assessment

Study participants themselves, or assisted by their parents, weighed all foods and beverages consumed as well as leftovers using electronic food scales to the nearest 1 g. In situations where weighing is not feasible, for example, out-of-home consumption, participants estimated their intakes semiquantitatively using common household measures (e.g., spoons, cups, portions). Participants provided information on specific food items, their brands, ingredients, and preparation. Trained dieticians entered the dietary records in the database after reviewing them for completeness and plausibility. Using a continuously updated in-house food composition database [26], food group intakes were determined. The nutritional content of staple foods was based on German food composition tables, while the energy and nutrient values of commercial food products were determined by recipe simulation.

Four food groups were included in this analysis: SSB included a diverse group of nondairy beverages with AS, including sweetened fruit juice drinks, sodas, sport energy drinks, and other flavored, carbonated, and noncarbonated soft drinks. LNCSB included aforementioned beverages but artificially sweetened with low- or no-caloric sweeteners, without AS. SBs included both SSB and LNCSB. AS included all sugars added to foods either during processing or manufacturing or during preparation or at the table [27]. The individual average of food intake from 3 recording days in grams per day was calculated.

#### Anthropometric measurements

Experienced nurses conducted different sets of anthropometric measurements. BMI (in kg/m^2^) and WC (centimeters) were determined by standard procedures. In children, BMI standard deviation scores (SDSs) were calculated using the national age- and sex-specific BMI percentiles as per Kromeyer–Hauschild method [28]. %BF was estimated from 4 skinfold thickness measurements (biceps, triceps, iliaca, and scapula), following age- and sex-specific equations by Deurenberg et al. [29]. Anthropometric measurements for this analysis were taken on, or nearest to, the date of urine collection and blood draw.

#### Other covariates assessment

Habitual leisure time physical activity was assessed using a questionnaire based on the Adolescent Physical Activity Recall Questionnaire [30], considering participation in organized (e.g., club sport, gym) and unorganized sports (e.g., cycling) over the previous year. Energy expenditure from these activities was quantified in metabolic equivalent of task hours per week. Self-reported smoking and alcohol status in adolescents and young adults was categorized into current, former, or never. Lifestyle factors (physical activity, smoking, and alcohol use) were assessed in adolescents and young adults only.

#### Urine samples

The 24-h urine samples were collected on the third day of their dietary assessment, following a standardized protocol. Within this period, urine was collected in preservative-free plastic containers and stored at less than −12°C until transferred to the study center where they were stored at −22°C until thawed and analyzed.

#### Blood samples

A fasting blood sample was drawn from participants and centrifuged at 4°C for 15 min (3100 U/min, 2000 G). Serum, plasma (citrate, EDTA) and buffy coat aliquots (500 μL each) were subsequently stored at −80°C. EDTA plasma was used in this study.

#### Metabolite profiling

Two independent laboratories, Metabolon and International Agency for Research on Cancer (IARC), performed untargeted metabolomics analysis, as shown in Figure 1. Metabolon used ultra–high-performance liquid chromatography-tandem mass spectroscopy to identify metabolites in adolescent urine and young adult plasma samples. Briefly, Metabolon carried out a set of standardized procedures from sample accession and preparation to analysis, raw data extraction and peak identification, following their internal standards [31]. In profiling the plasma samples, both metabolomics and lipidomics techniques were applied. In adolescent urine samples, 1407 features were annotated: 940 with known biochemical identity and 467 with no assigned chemical identity. In plasma samples, 1042 features were annotated: 811 with known chemical identity and 231 unknown.

IARC performed untargeted metabolite profiling using a ultra–high-performance liquid chromatography-tandem mass spectroscopy system (Q Exactive; Thermo Fischer Scinetific). Children urine samples (*n* = 600, representing 2 collections per participant at 2 time points) were analyzed next to each other in random order, and sample pairs were randomized across the batch. There were 4 independent analytical batches consisting of 2 individual 96-well plates. The mass spectrometer was operated in a positive/negative switching polarity. Preprocessing was performed using Compound Discoverer 3.3 software (Thermo Fisher Scientific) with minimum peak intensity threshold at 500,000, mass tolerance at 5 ppm, and feature alignment between samples performed with maximum retention time window of 0.05 min and mass tolerance of 5 ppm. Unlike Metabolon’s approach, metabolite features from IARC were first analyzed with dietary intake, and only features related to dietary intake were subsequently annotated. A detailed description of the analytical, quality control standards, and annotation procedures for both laboratories, is provided in Supplemental Methods.

### Statistical analyses

#### Participant characteristics

We calculated the median (25% and 75% percentile) for continuous variables and count (percentage) for categorical variables.

#### Preprocessing metabolomics data

We excluded metabolites that had missing values in >30% of the consumers of each food group. Missing values were imputed with half of the minimum value observed within each batch, with the assumption of missing due to low concentration below the instrument’s detection limit. Subsequently, these were natural log-transformed and standardized to have a mean of zero and unit variance. We corrected analytical batch effects by *ber* bagging method using the ‘dbnorm’ R package [32].

#### Metabolites selection

We applied 3 machine learning (ML) methods to first select and validate food-related metabolites, acknowledging the high dimensionality of the data sets and correlation among metabolites. These were random forest (RF), partial least squares (PLS), and least absolute shrinkage and selection operator (LASSO) with a bagging strategy. The PLS and RF were implemented using the multivariate modeling with minimally biased variable selection in R algorithm, a statistical validation framework that integrates a recursive ranking and backward elimination of variables within a repeated double cross-validation scheme [33]. The models were tuned following author recommendations [33] and were repeated 50 times to identify a stable set of metabolites ranked based on their importance to predict the respective dietary variable. The LASSO models were implemented using a variable-selection oriented LASSO bagging algorithm, combining LASSO regression with bootstrap aggregating to enhance stability and robust selection of biomarkers [34]. We generated 1000 bootstrap samples from the original data sets, and LASSO models were fitted on each sample using 5-fold cross-validation, all other parameters as per author description [34]. We applied the curve elbow point method to detect sharp drops in the observed frequency of variable selection. Metabolites with selection frequencies at and above the last elbow point were retained, if more than 1 point existed. For downstream analyses, we considered only metabolites selected by ≥2 ML methods to reduce the likelihood of selecting metabolites due to noise or method-specific bias. An overview of these steps is provided in Supplemental Figure 2.

#### Associations of food groups with metabolites

We used multivariable linear regression and linear mixed-effects models to examine the association of dietary intake and individual metabolites, for cross-sectional and repeated measures, respectively. In all regression models, we regressed each metabolite on intake (grams per day) adjusting for age, sex, and energy intake. For adolescents and young adults, we further adjusted for lifestyle factors (physical activity, alcohol, and smoking status). In children urine samples, the linear mixed-effects models included a random intercept for each participant. Because of the analytic design of long-term dietary assessment, plasma models were additionally adjusted for the difference in time between dietary assessment and blood draw (i.e., difference = age at blood draw – mean age of dietary assessments) and the number of dietary assessments. To account for multiple testing, we applied the Benjamini–Hochberg procedure to control the false discovery rate at 5%.

#### Associations of food-related metabolites with anthropometric measurements

To assess the associations of the food-related metabolites and adiposity, separate linear regression and linear mixed-effects models were constructed for each adiposity measure (BMI, %BF, and WC). We modeled these as response variables and sets of food-related metabolites and covariates [age, sex, energy intake, birthweight, and time difference (in days) between biosample collection and anthropometric measurements, and additionally, in adolescents and young adults, physical activity, alcohol, and smoking status] as predictor variables. In children urine samples, a random intercept for each participant was specified. To assess multicollinearity of the predictor variables, we used the variance inflation factor, and whenever appropriate, removed redundant metabolites with variance inflation factor of >10 [35], progressively starting from the highest. Considering the strong correlation between anthropometric measurements, we applied the modified Bonferroni method [36] to adjust the significance level for multiple testing.

#### Missing covariates

We used the K-Nearest Neighbor algorithm to impute the missing values in birthweight, physical activity, alcohol, and smoking status, with 10 nearest neighbors based on nonmissing values in other covariates (sex, age, BMI, energy intake, birthweight, physical activity, and alcohol and smoking status) implemented in the VIM R package. All statistical analyses were conducted using R 4.1.3 (The R Foundation for Statistical Computing).

## Results

### Participant characteristics

The median ages at biosample collection were 7.0 y (T_1_) and 8.0 y (T_2_) for children, 18.0 y for adolescents, and 18.1 y for young adults. Sex distribution was 52.9% female for T_1_ and 51.5% for T_2_ in children; 49.0% for adolescents; and 55.4% for young adults (Table 1).

**TABLE 1:** Basic characteristics of the study population^1^

	Children urine				Adolescent urine		Young adult plasma	
	*n*	T_1_, *n* = 297	*n*	T_2_, *n* = 270	*n*	Urine, *n* = 339	*n*	Plasma, *n* = 195
Sex: female	297	157 (52.9)	270	139 (51.5)	339	166 (49.0)	195	108 (55.4)
Age at biosample collection (y)	297	7.0 (7.0, 7.2)	270	8.0 (8.0, 8.2)	339	18.0 (17.0, 18.1)	195	18.1 (18.1, 18.2)
BMI (kg/m^2^)	297	15.8 (15.0, 17.1)	270	16.2 (15.1, 17.5)	339	21.9 (19.9, 24.0)	195	22.2 (20.1, 24.5)
Body fat percentage	296	17.3 (14.7, 20.4)	270	17.5 (14.8, 21.1)	339	22.6 (18.4, 27.1)	195	23.6 (19.2, 28.1)
LNCSB (g/d)	297	0.0 (0.0, 1022.3)	270	0.0 (0.0, 443.3)	339	0.0 (0.0, 0.0.0)	195	0.0 (0.0, 54.2)
Sugar-sweetened beverage (g/d)	297	44.0 (0.0, 163.3)	270	55.8 (0.0, 166.9)	339	133.3 (0.0, 418.3)	195	124.9 (51.2, 324.4)
Total sweetened beverages (g/d)	297	66.7 (0.0, 198.3)	270	66.7 (0.0, 216.9)	339	166.7 (0.0, 508.3)	195	163.8 (72.8, 408.4)
Added sugar (g/d)	297	46.5 (33.7, 64.7)	270	49.9 (35.0, 72.3)	339	62.2 (35.7, 89.9)	195	62.2 (43.8, 82.2)
Added sugar (% energy)	297	12.5 (9.4, 16.3)	270	12.5 (9.1, 17.2)	339	11.6 (7.4, 16.0)	195	12.5 (9.6, 15.3)
TEI (kcal/d)	297	1527.3 (1310.0, 1736.2)	270	1635.2 (1402.2, 1840.3)	339	2126.9 (1748.5, 2582.1)	195	1978.1 (1697.0, 2390.1)
Dietary assessments	297	1.0	270	1.0	339	1.0	195	4.0 (4.0, 5.0)
Physical activity (MET-h/w)	—	—	—	—	215	34.0 (14.1, 54.8)	184	30.1 (12.1, 52.9)
Smoking status	—	—	—	—	211		142	
Never	—	—	—	—	—	155 (73.5)	—	98 (69.0)
Former	—	—	—	—	—	23 (10.9)	—	21 (14.8)
Current	—	—	—	—	—	33 (15.6)	—	23 (16.2)
Alcohol status	—	—	—	—	179		153	
Never	—	—	—	—	—	24 (13.4)	—	20 (13.1)
Former	—	—	—	—	—	27 (15.1)	—	31 (20.3)
Current	—	—	—	—	—	128 (71.5)	—	102 (66.7)

Abbreviations: LNCSB, low- and no-calorie sweetened beverages; MET-h/w, metabolic equivalent of task-hours per week; TEI, total energy intake.

1 Data are given as *n* (%) and median (25%, 75%) for categorical and continuous variables, respectively. In children analytic sample, of the 297 participants in T_1_, 270 had repeated measures (T_2_). Although blood samples are collected at the age of 18 y or older, the dietary assessments in “young adults” mostly occurred during adolescence. Differences in *n* are due to missing data.

### Metabolite selections

There was good agreement in metabolite selections across the ML approaches. The PLS consistently selected more metabolites and shared common selections with the LASSO bagging algorithm, compared with the RF. The metabolite selections are provided for children urine, adolescent urine, and young adult plasma in Supplemental Tables 1–3, respectively.

### Associations of food groups with urine metabolites in children

LNCSB, SSB, SBs, and AS were associated with 4, 18, 18, and 28 metabolite features, respectively (Supplemental Table 4). Of the 8 biochemically identified metabolites, 7 associations were food specific (LNCSB positively associated with saccharin; SSB negatively associated with 4-pyridoxic acid; SBs negatively associated with kynurenic acid; and AS positively associated with theobromine, 7-methylguanine, aspartyphenylalanine, and negatively associated with uric acid). There was 1 common food-metabolite association: SSB, SBs, and AS were all associated with higher urinary concentrations of decadienoyl carnitine C10:2 (Table 2).

**TABLE 2:** Multivariable linear regression estimates of the associations of food groups with urine metabolites in children (*n* = 297)

Food	Metabolite	HMBD ID	β	95% CI	
				Lower	Upper
LNCSB	Saccharin	HMDB0029723	0.0016	0.0005	0.0027
SSB	Decadienoyl carnitine (C10:2)		0.0014	0.0009	0.0019
	4-pyridoxic acid	HMDB0000017	−0.0006	−0.0011	−0.0002
SBs	Decadienoyl carnitine (C10:2)		0.0013	0.0008	0.0017
	Kynurenic acid	HMDB0000715	−0.0008	−0.0013	−0.0003
AS	Decadienoyl carnitine (C10:2)		0.0120	0.0085	0.0155
	Theombromine	HMDB0002825	0.0080	0.0044	0.0116
	7-Methylguanine	HMDB000089	0.0055	0.0020	0.0092
	Aspartylphenylalanine	HMDB0000706	0.0050	0.0016	0.0084
	Uric acid	HMDB0000289	−0.0038	−0.0072	−0.0003

Abbreviations: AS, added sugar; CI, confidence intervals; HMBD ID, human metabolome database identification; LNCSB, low- and no-calorie sweetened beverages; SB, total sweetened beverage; SSB, sugar-sweetened beverage.

Models adjusted for age, sex, and energy intake, with a random intercept for each participant. Only biochemically identified metabolites with false discovery rate–adjusted *q* value <0.05 are shown. Complete list is given in Supplemental Table 1.

### Associations of food groups with urine metabolites in adolescents

**FIGURE 2:**
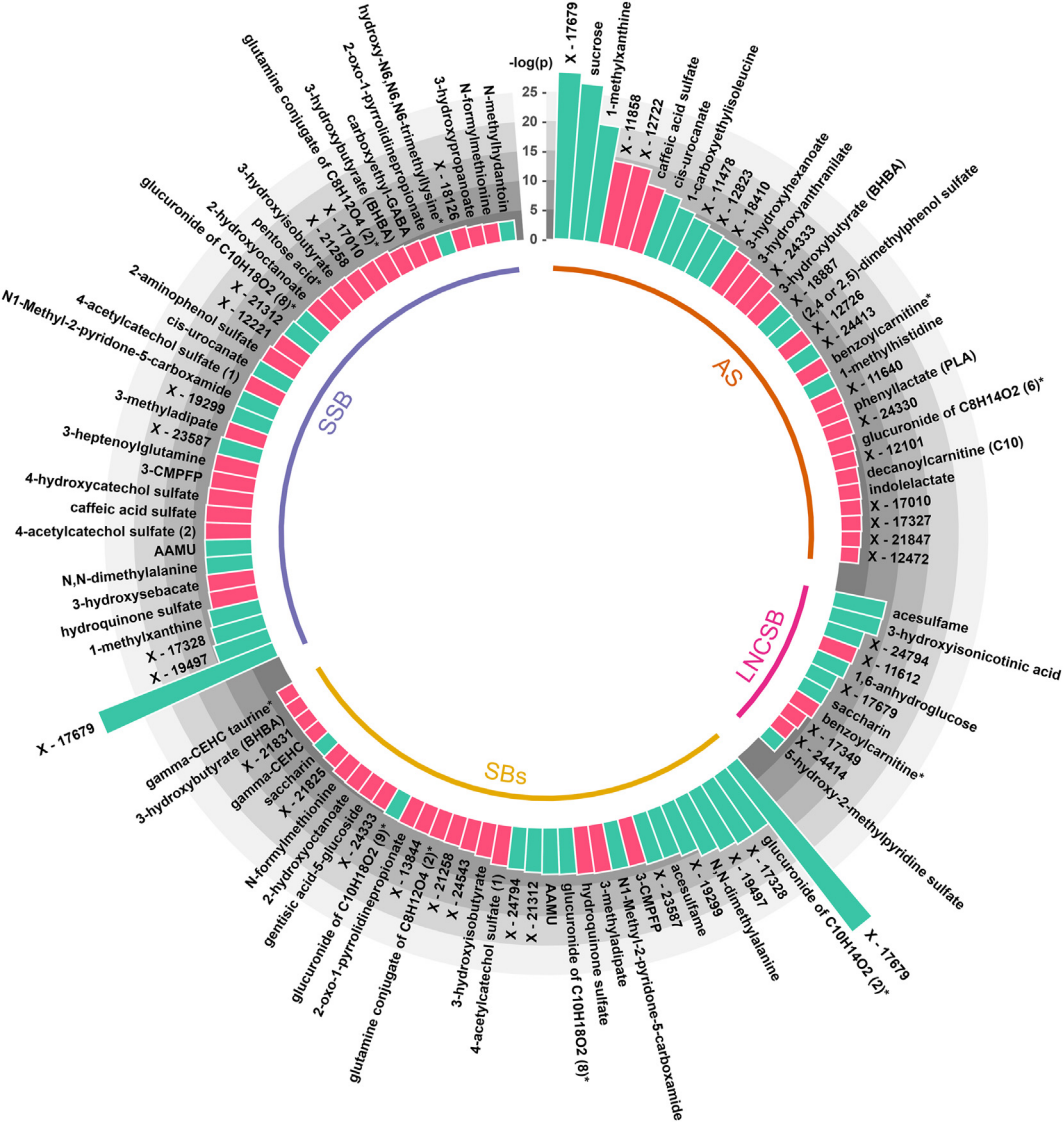
The associations of food groups with urine metabolites in adolescents. All models were adjusted for age, sex, energy intake, physical activity, alcohol and smoking status. Metabolites with false discovery rate–adjusted *q* value <0.05: AS, *n* = 32; LNCSB, *n* = 11; SSB, *n* = 37, and SBs, *n* = 34. The histogram bars represent the log-transformed *P* values: turquoise, positive association; light red, negative. ∗Metabolites not confirmed based on authentic standard, but Metabolon are confident in its identity. AS, added sugar; LNCSB, low- and no-calorie sweetened beverage; SB, total sweetened beverage; SSB, sugar-sweetened beverage.

Figure 2 summarizes metabolites associated with SBs and AS. LNCSB intake was associated with 11 metabolites (6 of them were specific to LNCSB), SSB intake with 37 metabolites (12 specific), SBs intake with 34 metabolites (9 specific), and AS intake with 32 metabolites (24 specific). All food-specific and nonspecific associations in adolescent urine samples can be found in Supplemental Table 5.

Briefly, LNCSB intake was associated with higher concentrations of acesulfame (β: 0.0012; 95% confidence interval [CI]: 0.0006, 0.0019) and saccharin (β: 0.0009; 95% CI: 0.0002, 0.0015). SSB intake was associated with higher concentrations of caffeine metabolites: 1-methylxanthine (β: 0.0005; 95% CI: 0.0003, 0.0008) and 5-acetylamino-6-amino-3-methyluracil (AAMU; β: 0.0005; 95% CI: 0.0002, 0.0008). Notably, SSB and SB intakes were also associated with elevated concentrations of unknown metabolites X-17679 (β: 0.0010; 95% CI: 0.0008, 0.0013; and β: 0.0010; 95% CI: 0.0007, 0.0012); X-19497 (β: 0.0005; 95% CI: 0.0003, 0.0008; and β: 0.0005; 95% CI: 0.0003, 0.0008); and X-17328 (β: 0.0005; 95% CI: 0.0003, 0.0008; and β: 0.0006; 95% CI: 0.0003, 0.0008), respectively. Other noteworthy associations included N1-methyl-2-pyridone-5-carboxamide (2PYr) with SSBs (β: 0.0004; 95% CI: 0.0002, 0.0007) and SBs (β: 0.0004; 95% CI: 0.0002, 0.0007) and N,N-dimethylalanine with SSBs (β: 0.0005; 95% CI: 0.0002, 0.0007) and SBs (β: 0.0005; 95% CI: 0.0003, 0.0008). AS intake was associated with higher concentrations of sucrose (β: 0.0095; 95% CI: 0.0069, 0.0121), X-17679 (β: 0.0098; 95% CI: 0.0073, 0.0124), and 1-methylxanthine (β: 0.0084; 95% CI: 0.0057, 0.0111).

### Associations of food groups with plasma metabolites

The associations of SBs and AS intakes with plasma metabolites are provided in Table 3. LNCSB intake was associated with 3 metabolites (2 of them specific to LNCSB intake), SSB with 11 metabolites (5 specific), SBs with 15 metabolites (8 specific), and AS with 3 metabolites (1 specific). Notably, 1-methyxanthine and AAMU were positively associated with SSBs (β: 0.0010; 95% CI: 0.0004, 0.0015; and β: 0.0009; 95% CI: 0.0003, 0.0014); SBs (β: 0.0010; 95% CI: 0.0005, 0.0015; and β: 0.0008; 95% CI: 0.0003, 0.0013); and AS (β: 0.0089; 95% CI: 0.0029, 0.0150; and β: 0.0091; 95% CI: 0.0031, 0.0151), respectively. Moreover, SSBs and SBs were associated with higher concentrations of caffeine (β: 0.0010; 95% CI: 0.0004, 0.0015; and β: 0.0009; 95% CI: 0.0005, 0.0014) and 1-3-dimethylurate (β: 0.0009; 95% CI: 0.0003, 0.0014; and β: 0.0008; 95% CI: 0.0004, 0.0013), respectively. All food-specific and nonspecific associations in plasma can be found in Supplemental Table 6.

**TABLE 3:** Multivariable linear regression estimates of the associations of food groups with plasma metabolites (*n* = 195)

Food	Metabolite	HMBD ID	β	95% CI	
				Lower	Upper
LNCSB	Octadecanedioylcarnitine (C18-DC)^1^		−0.0022	−0.0034	−0.0009
	Adipoylcarnitine (C6-DC)	HMDB61677	−0.0022	−0.0035	−0.0008
	3-bromo-5-chloro-2,6-dihydroxybenzoic acid^1^		0.0020	0.0007	0.0033
SSB	1-methylxanthine	HMDB10738	0.0010	0.0004	0.0015
	Caffeine	HMDB01847	0.0010	0.0004	0.0015
	1,3-dimethylurate	HMDB01857	0.0009	0.0003	0.0014
	AAMU	HMDB04400	0.0009	0.0003	0.0014
	X-16087		−0.0008	−0.0014	−0.0003
	3-CMPFP	HMDB61643	−0.0008	−0.0014	−0.0002
	X-13866		−0.0007	−0.0013	−0.0002
	Cyclopropyl 10:1 fatty acid (1)^1^		−0.0007	−0.0013	−0.0001
	Carotene diol (1)		−0.0006	−0.0011	−0.0001
	4-cholesten-3-one	HMDB00921	−0.0007	−0.0013	−0.0001
	X-24669		0.0007	0.0001	0.0012
SBs	1-methylxanthine	HMDB10738	0.0010	0.0005	0.0015
	Caffeine	HMDB01847	0.0009	0.0005	0.0014
	AAMU	HMDB04400	0.0008	0.0003	0.0013
	1,3-dimethylurate	HMDB01857	0.0008	0.0004	0.0013
	X-24951		0.0008	0.0003	0.0012
	X-16087		−0.0007	−0.0012	−0.0003
	X-24337		0.0007	0.0003	0.0012
	3-CMPFP	HMDB61643	−0.0008	−0.0012	−0.0003
	X-11308		0.0007	0.0002	0.0012
	Hydroquinone sulfate	HMDB02434	−0.0007	−0.0012	−0.0002
	X-17340		−0.0006	−0.0010	−0.0001
	N-formylphenylalanine		−0.0006	−0.0011	−0.0001
	Dihomo-linolenoylcarnitine (C20:3n3 or 6)^1^		0.0005	0.0001	0.0009
	Adipoylcarnitine (C6-DC)	HMDB61677	−0.0006	−0.0011	−0.0001
	Glutamine conjugate of C6H10O2 (1)^1^		−0.0006	−0.0010	−0.0001
AS	Etiocholanolone glucuronide	HMDB04484	−0.0115	−0.0177	−0.0053
	AAMU	HMDB04400	0.0091	0.0031	0.0151
	1-methylxanthine	HMDB10738	0.0089	0.0029	0.0150

Abbreviations: 3-CMPFP, 3-carboxy-4-methyl-5-pentyl-2-furanpropionate; AAMU, 5-acetylamino-6-amino-3-methyluracil; AS, added sugar; HMBD ID, human metabolome database identification; LNCSB, low- and no-calorie sweetened beverage; SB, total sweetened beverage; SSB, sugar-sweetened beverage.

All models adjusted for age, sex, energy intake, physical activity, alcohol and smoking status, number of dietary assessments, and the difference in time between dietary assessment and blood draw. Only metabolites with false discovery rate–adjusted *q* value < 0.05 are shown. The identities of X-, followed by a number (e.g., X-16087), are unknown.

1 Metabolites not confirmed based on an authentic standard, but Metabolon are confident in its identity.

### Associations of food-related metabolites with anthropometric measures

In children, 4 AS-related metabolite features, but of unknown biochemical identities, had mixed associations with BMI SDS and %BF (Table 4). One metabolite feature “214.08427@3.876” was positively associated with both BMI SDS (β: 0.08; 95% CI: 0.01, 0.014) and %BF (β: 0.59; 95% CI: 0.16, 1.02).

**TABLE 4:** Associations of food-related metabolites with adiposity measures

Food-metabolite	β	95% CI		*P*	β	95% CI		*P*	β	95% CI		*P*
		Lower	Upper			Lower	Upper			Lower	Upper	
Children urine	BMI SDS, *n* = 297				%BF, *n* = 297				WC			
165.07939@2.148	0.04	0.01	0.08	0.0092^1^	0.06	−0.17	0.30	0.5927	—	—	—	—
214.08427@3.876	0.08	0.01	0.14	0.0156^1^	0.59	0.16	1.02	0.0078^1^	—	—	—	—
153.04277@2.289	−0.03	−0.06	0.00	0.0515	−0.28	−0.51	−0.05	0.0176^1^	—	—	—	—
166.04911@1.902	−0.10	−0.18	−0.01	0.0293	−0.77	−1.39	−0.15	0.0158^1^	—	—	—	—
Adolescent urine	BMI, *n* = 339				%BF, *n* = 339				WC, *n* = 231			
X-24333	1.31	0.70	1.92	<0.0001^1^	1.10	0.25	1.95	0.0113^1^	2.31	0.20	4.42	0.0321
Acesulfame	0.82	0.43	1.22	0.0001^1^	0.92	0.36	1.47	0.0013^1^	1.40	0.10	2.70	0.0352
2PYr	0.63	0.21	1.05	0.0034^1^	0.86	0.27	1.44	0.0042^1^	1.98	0.64	3.32	0.0040^1^
N,N-dimethylalanine	−0.61	−1.03	−0.18	0.0055^1^	−1.18	−1.78	−0.58	0.0001^1^	−2.27	−3.66	−0.89	0.0014^1^
X-17679	−0.67	−1.20	−0.14	0.0128^1^	−0.41	−1.15	0.33	0.2721	−2.47	−4.15	−0.80	0.0040^1^
X-17010	−0.56	−1.00	−0.11	0.0139^1^	−0.63	−1.25	−0.01	0.0470	−1.13	−2.56	0.30	0.1198
X-17328	0.51	0.09	0.93	0.0176^1^	0.59	−0.00	1.18	0.0504	1.13	−0.16	2.43	0.0856
Decanoylcarnitine (C10)	1.09	0.62	1.56	<0.0001^1^	1.56	0.92	2.20	<0.0001^1^	4.41	2.85	5.96	<0.0001^1^
3-hydroxyhexanoate	−0.96	−1.49	−0.42	0.0005^1^	−1.43	−2.15	−0.71	0.0001^1^	−3.14	−4.86	−1.42	0.0004^1^
γ-CEHC taurine^2^	−1.15	−1.89	−0.40	0.0026^1^	−1.57	−2.58	−0.56	0.0024^1^	−2.65	−4.90	−0.41	0.0209
X-18887	−0.77	−1.36	−0.18	0.0103^1^	−0.67	−1.47	0.13	0.1018	−2.15	−3.92	−0.37	0.0179^1^
Glucuronide of C8H14O2 (6)^2^	0.57	0.02	1.12	0.0422	1.17	0.43	1.91	0.0022^1^	2.44	0.68	4.20	0.0069^1^
X-24330	0.52	0.02	1.02	0.0434	0.85	0.16	1.53	0.0152^1^	0.77	−0.93	2.46	0.3731
X-13844	−0.30	−0.78	0.18	0.2210	−0.79	−1.44	−0.13	0.0188^1^	−1.33	−2.79	0.14	0.0751
Cis-urocanate	−0.41	−0.82	−0.00	0.0488	−0.21	−0.78	0.36	0.4678	−1.55	−2.82	−0.29	0.0164^1^
Young adult plasma	BMI, *n* = 195				%BF, *n* = 195				WC, *n* = 195			
X-17340	1.02	0.31	1.74	0.0053^1^	1.26	0.26	2.26	0.0135^1^	2.05	0.31	3.79	0.0209
X-24337	0.76	0.18	1.33	0.0101^1^	0.42	−0.38	1.22	0.2991	1.52	0.13	2.92	0.0326
Carotene diol (1)	−0.67	−1.27	−0.07	0.0279	−0.73	−1.57	0.10	0.0855	−2.04	−3.50	−0.58	0.0063^1^

Abbreviations: %BF, body fat percentage; 2PYr, N1-methyl-2-pyridone-5-carboxamide; SDS, standard deviation score; WC, waist circumference.

Children urine: Adjusted for age, sex, energy intake, birthweight, and time difference between biosample collection and anthropometric measurements (in their original scale). WC measurements not available. Adolescent urine included all confounder adjustments for children samples, plus physical activity, smoking, and alcohol status. Young adult plasma included all confounder adjustments for adolescent urine samples, plus time difference between dietary assessment and blood draw and number of dietary assessments. The identities of X, followed by a number (e.g., X-24333), and the format “165.07939@2.148” are unknown.

1 Significant results: children urine, *P* < 0.0211; adolescent urine, *P* < 0.0205; and young adult plasma *P* < 0.0199 (modified Bonferroni method). Only food-related metabolites considered significant with either of the adiposity measures are shown.

2 Metabolites not confirmed based on authentic standard, but Metabolon are confident in its identity.

In adolescent urine samples, acesulfame was positively associated with BMI (β: 0.82; 95% CI: 0.43, 1.22) and %BF (β: 0.92; 95% CI: 0.36, 1.47). 2PYr was positively associated with BMI (β: 0.63; 95% CI: 0.21, 1.05), %BF (β: 0.86; 95% CI: 0.27, 1.44), and WC (β: 1.98; 95% CI: 0.64, 3.32). Decanoylcarnitine (C10) was also positively associated with BMI (β: 1.09; 95% CI: 0.62, 1.56), %BF (β: 1.56; 95% CI: 0.92, 2.20), and WC (β: 4.41; 95% CI: 2.85, 5.96). Two metabolites were inversely associated with all adiposity measures: N,N-dimethylalanine with BMI (β: −0.61; 95% CI: −1.03, −0.18); %BF (β: −1.18; 95% CI: −1.78, −0.58); and WC (β: −2.27; 95% CI: −3.66, −0.89); and 3-hydroxyhexanoate with BMI (β: −0.96; 95% CI: −1.49, −0.42); %BF (β: −1.43; 95% CI: −2.15, −0.71); and WC (β: −3.14; 95% CI: −4.86, −1.42) (Table 4).

In young adult plasma samples, 2 unknown metabolites showed positive associations: X-17340 with BMI (β: 1.02; 95% CI: 0.31, 1.74) and %BF (β: 1.26; 95% CI: 0.26, 2.26); and X-24337 with BMI (β: 0.76; 95% CI: 0.18, 1.33) only. Carotene diol (1) was inversely associated with WC (β: −2.04; 95% CI: −3.50, −0.58) (Table 4).

### Post-hoc exploration and analysis of bias

We sought to understand the overlapping of caffeine-related metabolites with SSB and AS across biosamples and whether these associations were confounded by other dietary sources of caffeine, such as coffee, chocolate, and other powdered instant beverages, including tea. Indeed, caffeine and caffeine-related metabolites measured in our study were associated with coffee intake, further providing plausibility of our results (Supplemental Table 7), but the main findings were robust to further adjustment with these foods (Supplemental Table 8). Next, we determined the correlation between SSB and AS intake and found a moderately strong Pearson correlation in adolescent urine (*r*: 0.65; 95% CI: 0.58, 0.71) and in young adult plasma (*r*: 0.78; 95% CI: 0.72, 0.83) samples.

Further, we examined potential bias due to half-minimum imputation of metabolite data in our analysis, as suggested previously [[37], [38], [39]]. We compared our results with those from quantile regression imputation, argued to be most optimal for limit of detection missingness [37] and RF imputation, favored for missing completely at random [39]. We observed comparable results across these imputation methods (Supplemental Figure 3).

Finally, we investigated the robustness of the observed associations of food-related metabolites with adiposity in adolescent urine and plasma using a different approach. For each anthropometric measurement, confounder-adjusted food-related metabolites were jointly fit in adaptive elastic-net regularized linear regression models as described previously [40]. We demonstrate that our main findings were robust and invariant to statistical modeling approach (Supplemental Tables 9 and 10).

## Discussion

This epidemiologic investigation, using 3 analytic data sets and both urine and plasma samples, identified robust metabolomics biomarkers of SBs and AS. In this study, we confirmed some previously reported metabolite biomarkers of SBs and AS and, to our knowledge, uncovered new ones that are robust across analytic samples. We also observed food-related metabolites that were consistently related to multiple anthropometric measures of adiposity.

Our children and adolescent data showed that urinary acesulfame and saccharin reflect LNCSB intake, with saccharin robust in both analytic samples. Given that LNCSB represent one of the main dietary sources of artificial sweeteners [41], acesulfame and saccharin are plausible urinary metabolite biomarkers of LNCSB intake. The 2 metabolites share similar biochemical properties, including absorption, distribution, metabolism, and excretion [42,43]. We did not detect the other artificial sweeteners in our urine samples. Their specific metabolism and excretion pathways may explain this result: metabolism into other compounds diluted in a large plasma/urine pool (e.g., aspartame into aspartic acid and phenylalanine), not detected by our analytical methods (e.g., steviol glycosides into glucuronides), or not absorbed in the gut (e.g., sucralose) [42,43]. Taken together, these results also suggest that our findings for other food-related metabolites are unlikely to be spurious and their relationship is metabolically plausible.

Moreover, our results indicated that caffeine metabolites, particularly 1-methylxanthine and AAMU, are consistently associated with SSB intake in adolescent urine and plasma samples, independent of all other plausible sources of caffeine as shown in our sensitivity analysis. Our study, therefore, confirms the association of SSB intake and elevated concentrations of AAMU in plasma [44] and additionally reports its reflection in urine.

A previous study proposed that SSB ingredients or their combinations could be explored as potential biomarkers for SSB as a group or subtypes of SSBs [19]. Although caffeine is one of the main ingredients of most SSBs, and multiple caffeine metabolites were consistently uncovered in adolescent urine and plasma, an important question remains as to whether a single metabolite biomarker of caffeinated SSB is possible. Based on our findings, it appears that a more promising approach to advance this science should consider combining metabolite biomarkers. We suggest that AAMU and 1-methylxanthine are promising urine and plasma metabolite biomarkers for caffeinated SSB and could be considered alongside other biomarkers such as isotopic signature δ^13^C or metabolite biomarkers of SSB ingredients such as taurine [21]. Besides, the SSBs are diverse, with varied concentrations of caffeine, taurine, and other ingredients, making it unlikely that a single, ingredient-based metabolite could reliably reflect overall SSB intake. The unknown biochemical compounds X-17679, X-19497, and X-17328, associated with SSB intake, represent an additional challenge.

Our untargeted metabolomics approach also confirmed the well-established association between AS intake and 24-hour urinary sucrose, reported in targeted approaches [16,45,46]. Our AS variable reflects intakes from various dietary sources. Despite a substantial portion originating from SSB and sugary snacks (e.g., cakes, candies, and desserts), 24-h urinary sucrose does not discriminate specific sources and would not be an ideal biomarker for SSB. This limitation of urinary sucrose, as well as of the isotopic signature δ^13^C, is extensively discussed elsewhere [47].

Besides the aforementioned putative metabolite biomarkers of SBs and AS, we also uncovered other metabolomics profiles worth highlighting. In children, SSB, SBs, and AS intake correlated with higher concentrations of decadienoyl carnitine (C10:2), a medium-chain acyl-carnitine involved in energy metabolism pathways [48]. We note that medium-chain acyl-carnitines are increasingly investigated as links to various metabolic dysfunctions [[48], [49], [50]] and depression [51]. To our knowledge, the association of C10:2 with SBs and AS intake has not been reported, but elevated concentrations of C10:2 with pork intake have been described [52].

The association between AS and aspartylphenylalanine may reflect the biochemical conversion of aspartame into aspartyl, phenylalanine, and methanol [53], and could indirectly relate to the positive correlation between SBs (sweetened with aspartame) and AS intakes as shown in our sensitivity analysis. The underlying mechanism of AS intake and elevated urinary 7-methylguanine, a biomarker of DNA damage and metabolic rate [54] is unclear. However, in another study, higher concentrations of 7-methylguanine were associated with unhealthy dietary habits [54].

Lower concentrations of kynurenic acid with intake of certain foods, such as SBs in our study, has been described in a longitudinal study [55], and this association has been observed in western-style dietary pattern [56]. Of note, kynurenic acid is an important metabolite of the tryptophan–kynurenine pathway, which is involved in modulation of inflammation and oxidative stress [57].

In adolescent urine and young adult plasma samples, SBs were associated with lower concentrations of 3-carboxy-4-methyl-5-pentyl-2-furanpropanoic acid, a metabolite of furan fatty acids. Humans acquire dietary furan fatty acids mainly from fish and fish oil [58] and are metabolized into 2 major metabolites: 3-carboxy-4-methyl-5-pentyl-2-furanpropanoic acid and 3-carboxy-4-methyl-5-propyl-2-furanpropanoic acid. Our findings across biosamples are therefore of interest, considering the important role furan fatty acids and health [[58], [59], [60]]. It is unclear whether there exists any interaction between SBs and furan fatty acid metabolism or this association is simply because dietary patterns characterized by higher intakes of SBs and AS correlate with overall poor diet quality [61,62]. In parallel, in adolescent urine and young adult plasma samples, we also observed an inverse association between SBs and hydroquinone sulfate, a specific marker of pear intake [63]. Pears are rich in dietary fibers, antioxidative flavonoids, and anti-inflammatory properties [64].

Regarding food-metabolite associations with adiposity, 4 metabolites in children urine samples showed mixed direction of associations with BMI and %BF. Their biochemical identities could not be identified. In adolescent urine samples, acesulfame was positively associated with both BMI and %BF. Acesulfame is not only a common sweetener for beverages but also added in confectionery, sweet, and savory snacks. Our sensitivity analysis showed poor correlation between acesulfame and self-reported intakes of these food items. Thus, we considered that LNCSBs were the likely primary source. This association could also suggest reverse causation and residual confounding, wherein individuals consuming LNCSB may already be overweight, and their beverage choices may be motivated by the intention to lose weight or to restrict their energy intake [65].

Similarly, the 2PYr concentrations were elevated with higher SSB and SBs intakes and positively with BMI, %BF, and WC. SBs are fortified with niacin, whose main metabolites are 2PYr and N-1-methylnicotinamide. Beneficial effects of niacin include neuroprotection, anti-inflammation, and immune modulation [66]. However, short-term metabolic effects of overconsumption of fortified beverages, such as glucose metabolism insulin secretion, have been observed in adolescents [67]. Their long-term effects on adiposity warrant further investigation.

Decanoylcarnitine (C10), positively associated with all adiposity measures in our study, is one of the medium-chain acyl-carnitines linked to body weight [68]. N,N-dimethylalanine and 3-hydroxyhexanoate were inversely associated with all adiposity measures; however, their biological basis remains unclear.

In plasma, carotene diol, a marker of leafy green and cruciferous vegetable intake [20], showed an inverse association with WC. This is consistent with findings from a large cohort study, where serum carotenoids correlated negatively with visceral adiposity [69]. We note that carotenoids are involved in oxidative and lipid metabolism [69] and higher concentrations of carotenoids are favorable for metabolic health. The biological role of X-17340 (associated with higher BMI and %BF) and X-24337 (higher BMI) are unknown.

This study also contributes to the public health discourse on caffeine and sugar pairing and health risks [70,71], by showing that caffeine added to SBs is also reflected at molecular level. Two randomized controlled trials showed that co-ingestion of carbohydrate load and caffeine impaired glucose and insulin responses in young, healthy males [72] and caffeine-containing energy drinks and shots resulted in acute impaired glucoregulation in healthy adolescents [73]. It appears that regular pairings of sugar and caffeine through SBs may influence adiposity through some of these mechanisms. Indeed, a recent study based on 3 large cohorts found that drinking unsweetened coffee, may prevent weight gain, but this benefit was negated by adding sugar [74].

A key strength of our study lies in the dynamic DONALD cohort design, which enables repeated dietary and biosample collections from the same individuals. The repeatedly measured metabolome in children uncovered potentially transient diet-metabolome associations, which may be missed in single point measurement. Our study applied multiple robust ML approaches, which generally yielded comparable selections; yet, the discrepancies also underscore the drawbacks of single-method reliance in high-dimensional data.

Further, the use of 3 standard adiposity measures, which assess the general and abdominal adiposity, enhances the translational utility and potential for our findings to be replicated by larger epidemiologic studies. To our knowledge, this study represents the first comprehensive exploration of the metabolome with SB and AS intakes and their associations with adiposity in young individuals. We demonstrate that our approach lends more insights, providing complimentary information on metabolic changes associated with intake, and their differences may reflect biologically meaningful processes.

Our study had some limitations such as potential measurement errors in self-reported dietary intakes. The interpretation of the associations of food-related metabolites and adiposity was limited by their concurrent measurements. Future studies may investigate the longitudinal associations of these metabolites with adiposity. We also acknowledge that, even with the repeated double cross-validation and bootstrap procedures, metabolite selection and the subsequent analysis were conducted on the same data set for maximum use, which could result in overly optimistic results in downstream regression analysis. The biochemical names of many metabolites in children samples could not be identified, limiting the comparison of our findings across age groups. Finally, the DONALD cohort’s homogeneity and higher socioeconomic status than the general population [25] warrants cautious interpretation of the results. Nonetheless, its adiposity trends from birth to 14 y are comparable with 2 other German cohorts [75], thus our findings have reasonable generalizability.

In conclusion, we identified metabolomics signatures of SB and AS intake and their associations with anthropometric measures of adiposity in a well-characterized German birth cohort. If validated in other studies, these metabolomics profiles could further elucidate the underlying mechanisms through which these foods influence adiposity.

## Author contributions

The authors’ responsibilities were as follows – UN, AF, MS, KO, SM: designed research; UA, IP, JG: conducted research; MM, JR, DA, AS, PK-R: provided essential materials; MES, SM: compiled dietary intake data; SM: analyzed data and wrote the first draft; KO: provided statistical supervision; SM, KO, UN: had primary responsibility for final content; and all authors: read and approved the final manuscript.

## Funding

This study was funded by the German Research Foundation (DFG 406710821) and the Agence Nationale de la Recherche and partly supported by Diet–Body–Brain (DietBB), the Competence Cluster in Nutrition Research (Federal Ministry of Education and Research, FKZ:01EA1410A), and the PerMiCCion project (Federal Ministry of Education and Research, Grant 100554612). KO is supported by the German Research Foundation (DFG 460591722).

## Data availability

The data described in the manuscript will be made available upon request, pending application and approval. Please submit your requests to Prof. Ute Nöthlings at epi@uni-bonn.de. The analytic code is available from the corresponding author.

## Conflict of interest

The authors report no conflicts of interests. Where authors are identified as personnel of the International Agency for Research on Cancer/WHO, the authors alone are responsible for the views expressed in this article and they do not necessarily represent the decisions, policy or views of the International Agency for Research on Cancer/WHO.
