# Supplementary material for: Metabolomics signatures of sweetened beverages and added sugar are related to anthropometric measures of adiposity in young individuals: results from a cohort study
Source: Am J Clin Nutr. 2024 Jul 24;120(4):879–90. doi: 10.1016/j.ajcnut.2024.07.021 (PMC11473401; doi:10.1016/j.ajcnut.2024.07.021)
Supplement: Multimedia component 1 [file mmc1.pdf]

Muli S, et al. Metabolomics signatures of sweetened beverages and added sugar are related to anthropometric measures of adiposity in young individuals: results from a cohort study

Correspondence : smuli@uni-bonn.de

Supplemental information

Table of contents

Supplemental Figure 1. Study population 2

Children Urine: IARC's Metabolomics Analysis Report 3

Adolescent Urine and young adult plasma: Metabolon's Metabolomics Analysis Report 4

Supplemental Figure 2. Metabolite section procedures 7

Supplemental Table 1. Machine learning selections of food-related metabolites in children (*n* = 297) 8

Supplemental Table 2. Machine learning selections of food-related metabolites in 'adolescent urine' (*n* = 339) 10

Supplemental Table 3. Machine learning selections of food-related metabolites in 'young adult plasma' (*n* = 195) 15

Supplemental Table 4. Metabolite features associated with SBs and AS intake in children (*n* = 297) 19

Supplemental Table 5: Metabolites associated with SBs and AS intake in 'adolescent urine' (*n* = 339) 20

Supplemental Table 6. Metabolites associated with SBs and AS intake in 'young adult plasma' (*n* = 195)........ 22

Supplemental Table 7: Associations of coffee intake with caffeine metabolites in 'adolescent urine' (n = 339) and 'young adult plasma' (n = 195) 23

Supplemental Table 8: Associations of SSB intake with all caffeine metabolites in 'adolescent urine' (n = 339) and 'young adult plasma' (n = 195) 24

Supplemental Figure 3. Assessing potential bias due to different imputation methods on food-metabolite associations 25

Supplemental Table 9. Food-related metabolites associated with adiposity using regularized regression in 'adolescent urine' 26

Supplemental Table 10. Food-related metabolites associated with adiposity using regularized regression 'young adult plasma' (n = 195) 28

References 29

Supplemental Figure 1. Study population

A

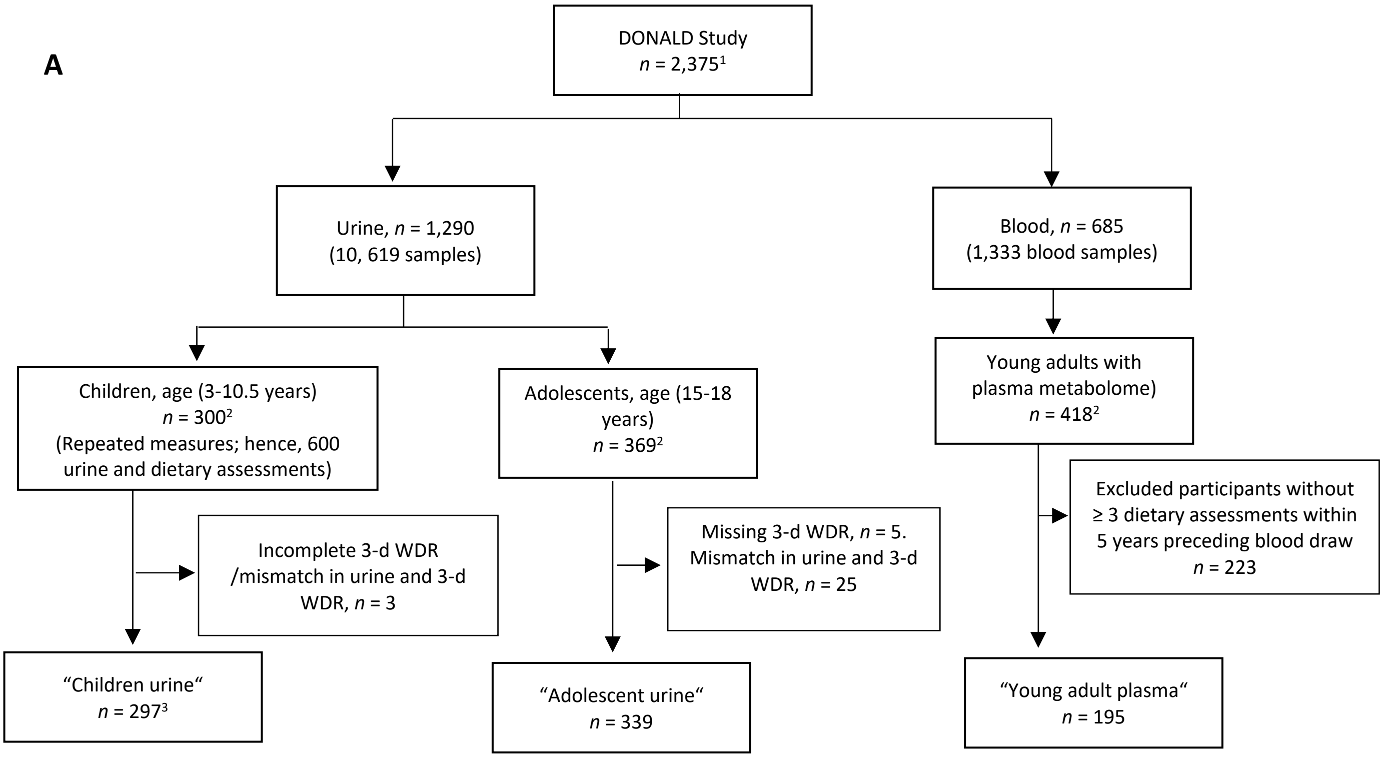

B

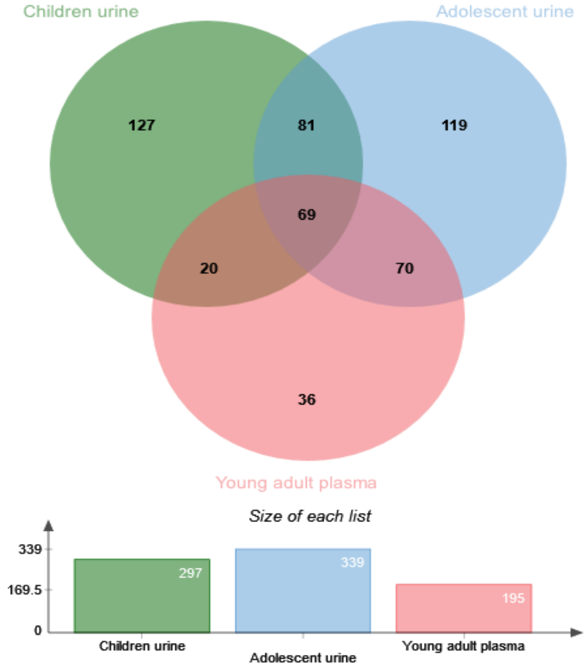

Supplemental Figure 1. (**A**): Flow diagram of the DONALD study samples included in the present analysis. [1]participants recruited between 1985 and December 2022. [2] these three samples were randomly selected among eligible urine and blood samples. [3] *n* = 297 of the 300 who provided two urine samples and dietary assessment were eligible (referred to as $\mathbf{T_1}$ in the manuscript). Out of these, 270 participants were eligible for inclusion as $\mathbf{T_2}$, representing the second dietary and urine measurement. Therefore, the "children urine" sample consisted of *n* = 567 urine and dietary samples that were jointly analyzed following appropriate methods for repeated measurements. (**B**): Overlap of the study participants across the analytic samples.

Children Urine: IARC's Metabolomics Analysis Report

Sample Preparation

Samples were prepared by diluting 30 µL of urine with ultra-pure water based on normalization to lowest specific gravity in all samples (DONALD: 1.079). Then 30 uL of the diluted urine samples were mixed with 270 µL of cold acetonitrile in Agilent Captiva 96 Deep Well plates (Agilent Technologies France; ref: A696001000B). The precipitate was filtered and 100 uL was transferred to Thermo Well 96 plates (Thermo Electron SAS; ref: 6820-4100). The plate was immediately sealed with a rapid EPS adhesive plate sheet (BioChromato; ref: BC-REPS001) and analysed. Quality control (QC) samples were prepared from a sample pool that was made by mixing small aliquots of all samples and extracted along with the study samples. Blank samples were also prepared along the urine samples in an identical manner, only leaving out urine in the process. Each well plate included four individually prepared QCs and two blanks.

Sample Analysis

Samples were analysed as 4 independent analytical batches consisting of 2 individual 96-well plates. The repeated samples points were analysed next to each other in random order, and sample pairs were randomized across the batch. A UHPLC-QE-MS system was used that consisted of a Dionex UltiMate 3000 Binary LC system, and a Q-Exactive mass spectrometer with heated electrospray ionization (HESI-II) (Thermo Scientific). Samples were kept at 5°C and 2 µL was injected. An ACQUITY UHPLC HSS T3 column (2.1 × 100mm, 1.8 µm; Waters) was used at 45 °C and the mobile phase consisted of ultrapure water and LC-MS grade methanol, both with 0.05 % (v/v) of formic acid. The gradient profile was as follows: 0–6 min: 5% to 100% methanol, 6–10.5 min: 100% methanol, 10.5–13 min: 5% methanol. The flow rate was 0.4 ml/min.

The mass spectrometer was operated in a positive/negative switching polarity using the following conditions: spray voltage 4.0 kV, sheath gas flow rate 50 (Arbitrary unit; A.u), auxillary gas flow rate 13 (A.u), sweep gas flow rate 3 (A.u), Aux gas heater temperature 425°C, capillary temperature 260°C and a S-Lens RF level 60%. For the analysis a full MS scan mode over a mass range of 66.7 to 1000 Da, at a resolution 35000 with an associated scan rate at 2.1Hz. AGC target 1e6 and a maximum injection time 50 ms was applied. MS/MS analyses were performed on QC samples with an isolation width of 2.0 Da, in positive and negative modes at 3 normalized collision energies 30, 60 and 90. Data was acquired in centroid format.

Data Processing

Pre-processing was performed using Compound Discoverer 3.3 software (Thermo Fisher Scientific). A minimum peak intensity threshold and mass tolerance of 600 000 and 10 ppm respectively were used to find $[M+H]^+$ ions in positive mode data. A minimum peak intensity threshold and mass tolerance of 500 000 and 10 ppm respectively were used to find $[M-H]^-$ ions in negative mode data. Feature alignment between samples was performed with maximum retention time window of 0.05 min and mass tolerance of 5ppm. Features present in every blank sample were excluded, unless 5-fold greater in average intensity in samples. Peak areas were used as a measurement of intensity.

Quality Control

Quality control was performed using data from the QC samples. The assessment was based on the following attributes:

- Response stability: in chronological order, area median response of features found in all QC samples
- Response variability: distribution of MS features according to their Relative Standard Deviations (RSD%) of features found in all QC samples
- Response variability: RSD% of known compounds in all QC samples.

Identification of metabolites

The features indicated by the statistical analysis as significant were grouped by retention time (with a tolerance of 0.05 min) and intensity using Spearman correlation across all samples to help in finding features originating from the same compound, requiring a minimum of correlation coefficient of 0.75. The features were compared

with the in-house database of analytical standards with 10 ppm molecular weight and 0.25 min retention time tolerance and search of the m/z values against the Human Metabolome Database (HMDB) [1] with a 10 ppm mass tolerance, considering $[M + H]^+$, $[M + Na]^+$, and $[M - H_2O + H]^+$ adducts in positive mode and $[M - H]^-$, $[M + FA - H]^-$, and $[M - H_2O - H]^-$ in negative mode.

The quality of the chromatographic peaks and spectra was inspected, and the plausibility of database candidates was assessed based on retention time, isotope pattern, adduct formation and neutral losses. The best matching identities were confirmed by MS/MS spectra of standard MS/MS. When standards are not available, MS/MS spectra were compared against those in mzCloud (www.mzcloud.org) or METLIN (www.metlin.scripps.edu) [2]. The level of identification was determined as proposed by Sumner et al. [3].

Adolescent Urine and young adult plasma: Metabolon's Metabolomics Analysis Report

Metabolon Platform

Sample Accessioning: Following receipt, samples were inventoried and immediately stored at -80ºC. Each sample received was accessioned into the Metabolon LIMS system and was assigned by the LIMS a unique identifier that was associated with the original source identifier only. This identifier was used to track all sample handling, tasks, results, etc. The samples (and all derived aliquots) were tracked by the LIMS system. All portions of any sample were automatically assigned their own unique identifiers by the LIMS when a new task was created; the relationship of these samples was also tracked. All samples were maintained at -80ºC until processed.

Sample Preparation: Samples were prepared using the automated MicroLab STAR® system from Hamilton Company. Several recovery standards were added prior to the first step in the extraction process for QC purposes. To remove protein, dissociate small molecules bound to protein or trapped in the precipitated protein matrix, and to recover chemically diverse metabolites, proteins were precipitated with methanol under vigorous shaking for 2 min (Glen Mills GenoGrinder 2000) followed by centrifugation. The resulting extract was divided into five fractions: two for analysis by two separate reverse phase (RP)/UPLC-MS/MS methods with positive ion mode electrospray ionization (ESI), one for analysis by RP/UPLC-MS/MS with negative ion mode ESI, one for analysis by HILIC/UPLC-MS/MS with negative ion mode ESI, and one sample was reserved for backup. Samples were placed briefly on a TurboVap® (Zymark) to remove the organic solvent. The sample extracts were stored overnight under nitrogen before preparation for analysis.

QA/QC: Several types of controls were analyzed in concert with the experimental samples: a pooled matrix sample generated by taking a small volume of each experimental sample (or alternatively, use of a pool of well-characterized human plasma) served as a technical replicate throughout the data set; extracted water samples served as process blanks; and a cocktail of QC standards that were carefully chosen not to interfere with the measurement of endogenous compounds were spiked into every analyzed sample, allowed instrument performance monitoring and aided chromatographic alignment. Instrument variability was determined by calculating the median relative standard deviation (RSD) for the standards that were added to each sample prior to injection into the mass spectrometers. Overall process variability was determined by calculating the median RSD for all endogenous metabolites (i.e., non-instrument standards) present in 100% of the pooled matrix samples. Experimental samples were randomized across the platform run with QC samples spaced evenly among the injections

Ultrahigh Performance Liquid Chromatography-Tandem Mass Spectroscopy (UPLC-MS/MS): All methods utilized a Waters ACQUITY ultra-performance liquid chromatography (UPLC) and a Thermo Scientific Q-Exactive high resolution/accurate mass spectrometer interfaced with a heated electrospray ionization (HESI-II) source and Orbitrap mass analyzer operated at 35,000 mass resolution. The sample extract was dried then reconstituted in solvents compatible to each of the four methods. Each reconstitution solvent contained a series of standards at fixed concentrations to ensure injection and chromatographic consistency. One aliquot was analyzed using acidic positive ion conditions, chromatographically optimized for more hydrophilic compounds. In this method, the

extract was gradient eluted from a C18 column (Waters UPLC BEH C18-2.1x100 mm, 1.7 µm) using water and methanol, containing 0.05% perfluoropentanoic acid (PFPA) and 0.1% formic acid (FA). Another aliquot was also analyzed using acidic positive ion conditions, however it was chromatographically optimized for more hydrophobic compounds. In this method, the extract was gradient eluted from the same afore mentioned C18 column using methanol, acetonitrile, water, 0.05% PFPA and 0.01% FA and was operated at an overall higher organic content. Another aliquot was analyzed using basic negative ion optimized conditions using a separate dedicated C18 column. The basic extracts were gradient eluted from the column using methanol and water, however with 6.5mM Ammonium Bicarbonate at pH 8. The fourth aliquot was analyzed via negative ionization following elution from a HILIC column (Waters UPLC BEH Amide 2.1x150 mm, 1.7 µm) using a gradient consisting of water and acetonitrile with 10mM Ammonium Formate, pH 10.8. The MS analysis alternated between MS and data-dependent MS^n scans using dynamic exclusion. The scan range varied slighted between methods but covered 70-1000 m/z. Raw data files are archived and extracted as described below.

Bioinformatics: The informatics system consisted of four major components, the Laboratory Information Management System (LIMS), the data extraction and peak-identification software, data processing tools for QC and compound identification, and a collection of information interpretation and visualization tools for use by data analysts. The hardware and software foundations for these informatics components were the LAN backbone, and a database server running Oracle 10.2.0.1 Enterprise Edition.

LIMS: The purpose of the Metabolon LIMS system was to enable fully auditable laboratory automation through a secure, easy to use, and highly specialized system. The scope of the Metabolon LIMS system encompasses sample accessioning, sample preparation and instrumental analysis and reporting and advanced data analysis. All of the subsequent software systems are grounded in the LIMS data structures. It has been modified to leverage and interface with the in-house information extraction and data visualization systems, as well as third party instrumentation and data analysis software.

Data Extraction and Compound Identification: Raw data was extracted, peak-identified and QC processed using Metabolon's hardware and software. These systems are built on a web-service platform utilizing Microsoft's .NET technologies, which run on high-performance application servers and fiber-channel storage arrays in clusters to provide active failover and load-balancing. Compounds were identified by comparison to library entries of purified standards or recurrent unknown entities. Metabolon maintains a library based on authenticated standards that contains the retention time/index (RI), mass to charge ratio (*m/z)*, and chromatographic data (including MS/MS spectral data) on all molecules present in the library. Furthermore, biochemical identifications are based on three criteria: retention index within a narrow RI window of the proposed identification, accurate mass match to the library +/- 10 ppm, and the MS/MS forward and reverse scores between the experimental data and authentic standards. The MS/MS scores are based on a comparison of the ions present in the experimental spectrum to the ions present in the library spectrum. While there may be similarities between these molecules based on one of these factors, the use of all three data points can be utilized to distinguish and differentiate biochemicals. More than 3300 commercially available purified standard compounds have been acquired and registered into LIMS for analysis on all platforms for determination of their analytical characteristics. Additional mass spectral entries have been created for structurally unnamed biochemicals, which have been identified by virtue of their recurrent nature (both chromatographic and mass spectral). These compounds have the potential to be identified by future acquisition of a matching purified standard or by classical structural analysis.

Curation: A variety of curation procedures were carried out to ensure that a high quality data set was made available for statistical analysis and data interpretation. The QC and curation processes were designed to ensure accurate and consistent identification of true chemical entities, and to remove those representing system artifacts, mis-assignments, and background noise. Metabolon data analysts use proprietary visualization and interpretation software to confirm the consistency of peak identification among the various samples. Library matches for each compound were checked for each sample and corrected if necessary.

Metabolite Quantification and Data Normalization: Peaks were quantified using area-under-the-curve. For studies spanning multiple days, a data normalization step was performed to correct variation resulting from

instrument inter-day tuning differences. Essentially, each compound was corrected in run-day blocks by registering the medians to equal one (1.00) and normalizing each data point proportionately i.e., "block correction". For studies that did not require more than one day of analysis, no normalization is necessary, other than for purposes of data visualization. In certain instances, biochemical data may have been normalized to an additional factor (e.g., cell counts, total protein as determined by Bradford assay, osmolality, etc.) to account for differences in metabolite levels due to differences in the amount of material present in each sample.

Complex Lipids Platform: Adolescent Plasma

Lipids were extracted from samples in methanol:dichloromethane in the presence of internal standards. The extracts were concentrated under nitrogen and reconstituted in 0.25mL of 10mM ammonium acetate dichloromethane:methanol (50:50). The extracts were transferred to inserts and placed in vials for infusion-MS analysis, performed on a Shimazdu LC with nano PEEK tubing and the Sciex SelexIon-5500 QTRAP. The samples were analyzed via both positive and negative mode electrospray. The 5500 QTRAP scan was performed in MRM mode with the total of more than 1,100 MRMs. Individual lipid species were quantified by taking the peak area ratios of target compounds and their assigned internal standards, then multiplying by the concentration of internal standard added to the sample. Lipid class concentrations were calculated from the sum of all molecular species within a class, and fatty acid compositions were determined by calculating the proportion of each class comprised by individual fatty acids.

Supplemental Figure 2. Metabolite selection procedures

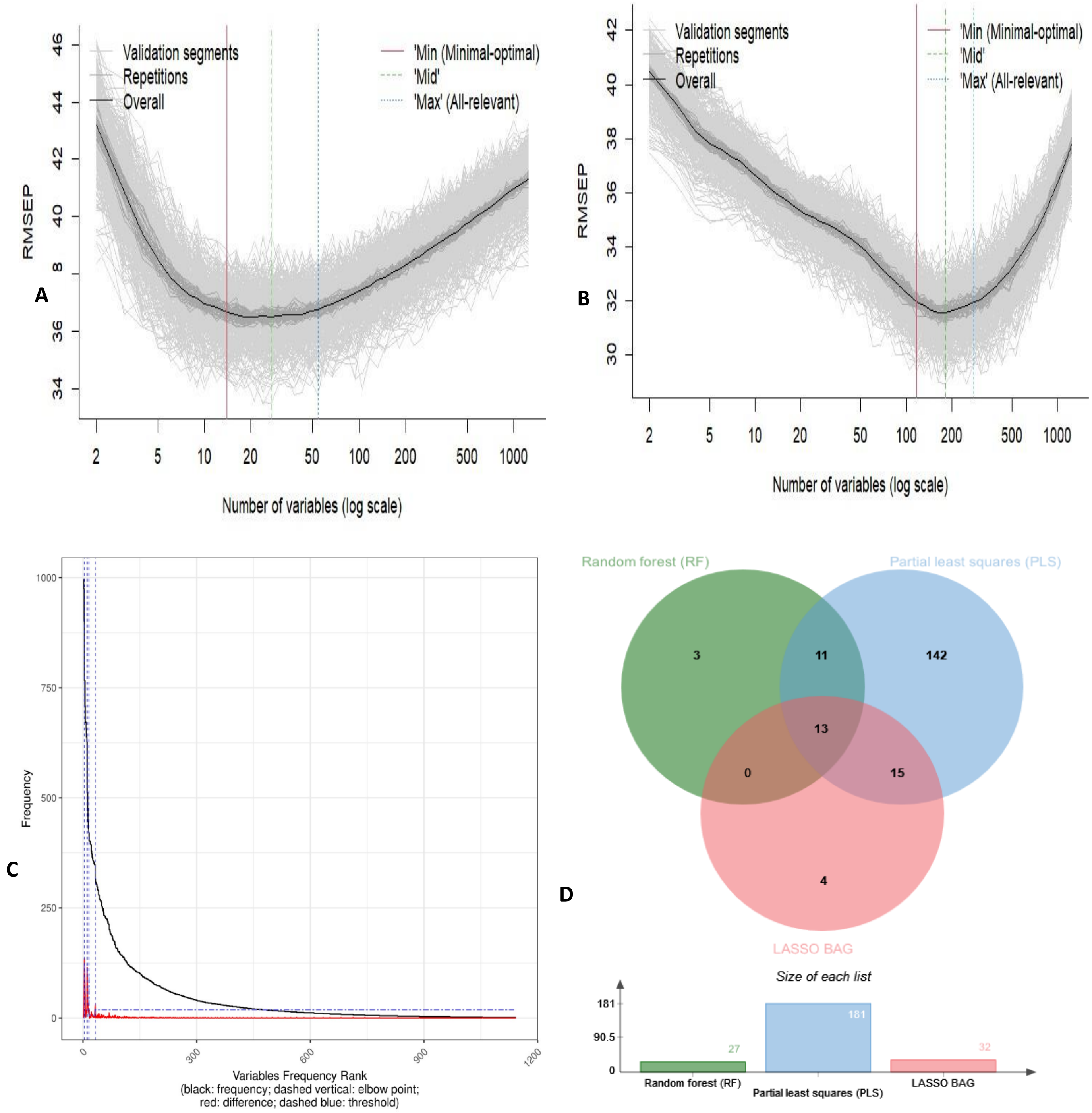

Supplemental Figure 2. An example of AS metabolites in urine adolescent (n = 339). **A** and **B**: Random forest (RF) and partial least squares (PLS) regression models, respectively. In both models, the light grey lines represent validation performance for the individual inner segments, while the darker grey lines represent inner segment validation curves averaged over the repetitions. Three model results are returned: Minimal-optimal (Min), Mid, and All-relevant (Max). The Min model selects the smallest set of metabolites that optimizes the method performance and identifies the strongest predictors of target variable Y (dietary intake). The Max model includes all the strongest predictors, including redundant, but not necessarily spurious. The Mid model is a trade-off between the Min and Max models, and was used for the present analysis. **C**: The "Curve Elbow Point" (CEP) detection method for LASSO bagging models. The most important metabolites are those with a high frequency of selection, separated from the rest by a sharp drop in the observed frequency. Metabolites at or above the elbow point were considered important of intake. **D**: The overlap of metabolite selection (for AS) across the models. Metabolites were selected by a "two out of three" ML approach, which effectively balances flexibility and reliability. Unless otherwise specified, we modelled all dietary variables (SSB, SBs, and AS) as continuous, and LNCSB, which had few consumers was dichotomized into consumers vs non-consumers.

Supplemental Table 1. Machine learning selections of food-related metabolites in children (*n* = 297)

**Metabolite feature**	**LNCSB**	**SSB**	**SBs**	**AS**
168.05358@1.786	RF, LASSO-BAG, PLS	-	RF, LASSO-BAG, PLS	-
182.99887@2.451	RF, LASSO-BAG, PLS	-	-	-
184.08475@1.608	RF, PLS	-	RF, LASSO-BAG, PLS	RF, PLS
198.1007@2.172	RF, PLS	-	-	-
233.03563@1.471	RF, PLS	-	RF, LASSO-BAG, PLS	RF, PLS
256.14245@3.937	RF, PLS	-	-	RF, PLS
121.91727@0.601	-	RF, LASSO-BAG, PLS	LASSO-BAG, PLS	-
128.0841@3.753	-	RF, LASSO-BAG, PLS	RF, PLS	-
129.96719@0.626	-	LASSO-BAG, PLS	-	-
134.10968@4.712	-	-	RF, PLS	-
136.03732@0.724	-	LASSO-BAG, PLS	-	-
142.02683@2.132	-	-	RF, LASSO-BAG, PLS	-
148.08901@3.889	-	RF, PLS	-	-
150.06827@4.068	-	-	-	RF, PLS
152.12024@4.714	-	RF, PLS	RF, PLS	-
153.04277@2.289	-	-	-	RF, LASSO-BAG, PLS
158.05809@2.816	-	-	-	RF, PLS
165.0652@1.186	-	-	-	LASSO-BAG, PLS
165.07939@2.148	-	-	-	RF, PLS
166.04911@1.902	-	-	-	RF, PLS
168.02863@0.967	-	-	-	LASSO-BAG, PLS
176.06858@3.179	-	-	RF, LASSO-BAG, PLS	-
180.06467@2.415	-	-	-	RF, PLS
183.05335@1.813	-	LASSO-BAG, PLS	-	-
189.04266@3.053	-	-	RF, PLS	-
195.05254@2.557	-	-	-	RF, PLS
214.08427@3.876	-	-	-	RF, PLS
214.12043@5.044	-	-	RF, PLS	-
217.09495@1.938	-	-	-	RF, LASSO-BAG
220.00374@1.773	-	LASSO-BAG, PLS	-	-
228.11091@0.776	-	-	-	RF, LASSO-BAG, PLS
230.12675@2.177	-	-	-	RF, LASSO-BAG, PLS
234.01955@2.357	-	-	-	RF, LASSO-BAG, PLS
242.12665@3.379	-	-	RF, LASSO-BAG, PLS	RF, LASSO-BAG, PLS
244.08815@2.104	-	-	-	RF, PLS
246.14676@4.666	-	-	-	LASSO-BAG, PLS
256.14227@3.906	-	RF, LASSO-BAG, PLS	RF, LASSO-BAG, PLS	RF, PLS
258.15795@4.241	-	-	RF, PLS	RF, LASSO-BAG, PLS
258.15815@4.273	-	-	LASSO-BAG, PLS	RF, PLS
259.08166@0.781	-	-	-	LASSO-BAG, PLS
259.99915@3.019	-	RF, LASSO-BAG, PLS	RF, LASSO-BAG, PLS	-
261.12113@0.936	-	-	LASSO-BAG, PLS	-
280.10589@2.888	-	-	-	LASSO-BAG, PLS
282.11048@4.718	-	LASSO-BAG, PLS	LASSO-BAG, PLS	-

284.0897@3.588	-	-	-	RF, PLS
290.09368@2.095	-	-	-	LASSO-BAG, PLS
301.11611@3.557	-	-	-	RF, PLS
311.20953@4.339	-	RF, PLS	RF, PLS	RF, PLS
342.1315@4.916	-	-	-	RF, PLS
345.21495@2.18	-	RF, LASSO-BAG, PLS	RF, LASSO-BAG, PLS	-
346.16262@3.85	-	RF, LASSO-BAG, PLS	RF, LASSO-BAG, PLS	RF, LASSO-BAG, PLS
346.16262@4.744	-	-	-	RF, PLS
346.16267@4.709	-	RF, PLS	-	-
358.10867@5.056	-	-	LASSO-BAG, PLS	-
364.09779@0.732	-	RF, PLS	RF, PLS	-
378.0928@0.759	-	RF, LASSO-BAG, PLS	LASSO-BAG, PLS	RF, LASSO-BAG, PLS
440.08768@0.759	-	-	-	RF, LASSO-BAG, PLS
453.19967@4.707	-	LASSO-BAG, PLS	-	-

Abbreviations: AS, added sugar; LASSO-BAG, Least Absolute Shrinkage and Selection Operator with bagging algorithm; LNCSB, low- and no-calorie sweetened beverages; PLS, partial least squares; RF, random forest; SBs, total sweetened beverages; SSB, sugar sweetened beverages.

Supplemental Table 2. Machine learning selections of food-related metabolites in 'adolescent urine' (*n* = 339)

metabolite	LNCSB	SSB	SBs	AS	HMDB ID	RI	Mass	Platform	Pubchem ID	superpathway
saccharin	RF, LASSO, PLS	-	LASSO, PLS	-	HMDB29723	2000	181.99173	LC/MS Neg	5143	Xenobiotics
X - 11612	RF, LASSO, PLS	-	-	-	-	1633	283.06849	LC/MS Neg	-	-
acesulfame	RF, LASSO, PLS	-	RF, LASSO, PLS	-	HMDB33585	1581	161.98665	LC/MS Neg	36573	Xenobiotics
X - 24794	RF, LASSO, PLS	-	RF, PLS	-	-	1131	123.05621	LC/MS Neg	-	-
3-hydroxyisonicotinic acid	RF, LASSO, PLS	-	-	-	-	1497	138.01966	LC/MS Neg	459503	Xenobiotics
3-hydroxybenzoate	RF, PLS	-	-	-	HMDB02466	1180	137.02442	LC/MS Polar	7420	Xenobiotics
1,6-anhydroglucose	LASSO, PLS	-	-	-	HMDB00640	1175.5	207.05102	LC/MS Polar	2724705	Xenobiotics
benzoylcarnitine*	LASSO, PLS	-	-	LASSO, PLS	-	3041	266.13869	LC/MS Pos Early	-	Xenobiotics
X - 17349	LASSO, PLS	-	-	-	-	2497	367.10412	LC/MS Neg	-	-
X - 17679	LASSO, PLS	RF, LASSO, PLS	RF, LASSO, PLS	RF, LASSO, PLS	-	1775	199.09797	LC/MS Neg	-	-
X - 24414	RF, PLS	-	-	-	-	1965	234.08027	LC/MS Neg	-	-
5-hydroxy-2-methylpyridine sulfate	LASSO, PLS	-	-	-	-	2074	188.0023	LC/MS Neg	-	Xenobiotics
sucrose	-	-	-	RF, LASSO, PLS	HMDB00258	865	341.10894	LC/MS Neg	5988	Carbohydrate
3-hydroxyisobutyrate	-	LASSO, PLS	LASSO, PLS	-	HMDB00336	1619	103.04007	LC/MS Polar	87	Amino Acid
3-hydroxypropanoate	-	LASSO, PLS	-	-	HMDB00700	1845	89.02442	LC/MS Polar	68152	Lipid
indolelactate	-	-	-	RF, LASSO, PLS	HMDB00671	2286	204.06661	LC/MS Neg	92904	Amino Acid
2-hydroxyoctanoate	-	RF, LASSO, PLS	RF, LASSO, PLS	-	HMDB02264	3736.8	159.10266	LC/MS Neg	94180	Lipid
phenyllactate (PLA)	-	-	-	LASSO, PLS	HMDB00779	908	165.05571	LC/MS Polar	3848	Amino Acid
alpha-hydroxyisocaproate	-	-	-	LASSO, PLS	HMDB00746	1840	131.07136	LC/MS Neg	83697	Amino Acid
N-formylmethionine	-	LASSO, PLS	LASSO, PLS	-	HMDB01015	1543.8	176.03869	LC/MS Neg	439750	Amino Acid
1-methylhistidine	-	-	-	LASSO, PLS	HMDB00001	1227	168.07785	LC/MS Neg	92105	Amino Acid

3-hydroxysebacate	-	LASSO, PLS	-	-	HMDB00350	2411	217.10814	LC/MS Polar	3017884	Lipid
3-hydroxyanthranilate	-	-	-	LASSO, PLS	HMDB01476	2407	154.04987	LC/MS Pos Early	86	Amino Acid
decanoylcarnitine (C10)	-	-	-	LASSO, PLS	HMDB00651	1130	316.24824	LC/MS Pos Late	10245190	Lipid
1-methylxanthine	-	LASSO, PLS	-	LASSO, PLS	HMDB10738	1568.8	165.0418	LC/MS Neg	80220	Xenobiotics
phosphocholine	-	-	-	LASSO, PLS	HMDB01565	700	184.07332	LC/MS Pos Early	1014	Lipid
AAMU	-	LASSO, PLS	LASSO, PLS	-	HMDB04400	1710	197.06801	LC/MS Polar	88299	Xenobiotics
5-methyluridine (ribothymidine)	-	-	-	RF, LASSO, PLS	HMDB00884	1778.1	257.07791	LC/MS Neg	445408	Nucleotide
hydroquinone sulfate	-	LASSO, PLS	LASSO, PLS	-	HMDB02434	1395	188.98631	LC/MS Neg	161220	Xenobiotics
3-methyladipate	-	RF, LASSO, PLS	RF, LASSO, PLS	-	HMDB00555	2865	159.06628	LC/MS Polar	12292	Lipid
N-carbamoylsarcosine	-	LASSO, PLS	-	-	HMDB12265	920	133.06077	LC/MS Pos Early	439375	Amino Acid
homocitrate	-	LASSO, PLS	-	LASSO, PLS	HMDB03518	1179	224.0765	LC/MS Pos Early	439459	Xenobiotics
N-methylhydantoin	-	LASSO, PLS	-	-	HMDB03646	1425	113.03565	LC/MS Neg	69217	Amino Acid
carboxyethyl-GABA	-	LASSO, PLS	-	-	HMDB02201	2085	176.09174	LC/MS Pos Early	2572	Amino Acid
cis-urocanate	-	LASSO, PLS	-	LASSO, PLS	HMDB34174	1200	137.03565	LC/MS Neg	1549103	Amino Acid
2-oxo-1-pyrrolidinepropionate	-	LASSO, PLS	LASSO, PLS	-	-	1600	158.08117	LC/MS Pos Early	3146688	Xenobiotics
2PYr	-	LASSO, PLS	RF, LASSO, PLS	-	HMDB04193	1668	151.0513	LC/MS Neg	69698	Cofactors and Vitamins
gamma-CEHC glucuronide*	-	RF, PLS	-	-	-	2500	439.16097	LC/MS Neg	-	Cofactors and Vitamins
2-aminophenol sulfate	-	RF, PLS	-	-	HMDB61116	1677	188.0023	LC/MS Neg	181670	Xenobiotics
gamma-CEHC	-	-	RF, PLS	-	HMDB01931	3843	263.12888	LC/MS Neg	133098	Cofactors and Vitamins
X - 21258	-	RF, LASSO, PLS	RF, LASSO	-	-	3915	213.02316	LC/MS Neg	-	-
X - 21312	-	LASSO, PLS	LASSO, PLS	-	-	2757.7	243.03368	LC/MS Neg	-	-
X - 11478	-	-	-	RF, PLS	-	4285	165.09214	LC/MS Neg	-	-
X - 12472	-	-	-	RF, PLS	-	2528	241.11935	LC/MS Neg	-	-

X - 21825	-	LASSO, PLS	LASSO, PLS	-	-	3220	234.07805	LC/MS Neg	-	-
X - 21831	-	-	RF, LASSO	-	-	3480	363.16759	LC/MS Neg	-	-
X - 21847	-	-	-	RF, LASSO, PLS	-	4270	403.10512	LC/MS Neg	-	-
X - 18887	-	-	-	RF, LASSO, PLS	-	2229	328.15182	LC/MS Neg	-	-
X - 18410	-	-	-	RF, PLS	-	1538	305.07041	LC/MS Neg	-	-
X - 11640	-	-	-	RF, PLS	-	3769	377.07052	LC/MS Neg	-	-
X - 12101	-	-	-	RF, PLS	-	2700	164.07386	LC/MS Pos Early	-	-
X - 11858	-	-	-	RF, LASSO, PLS	-	4414	437.0555	LC/MS Neg	-	-
X - 19299	-	LASSO, PLS	LASSO, PLS	-	-	3728	227.12896	LC/MS Neg	-	-
X - 19497	-	RF, LASSO, PLS	RF, LASSO, PLS	-	-	3643	227.12921	LC/MS Neg	-	-
X - 17327	-	-	-	RF, PLS	-	2750	257.14918	LC/MS Pos Early	-	-
X - 17328	-	LASSO, PLS	LASSO, PLS	-	-	910	308.18508	LC/MS Pos Late	-	-
X - 18126	-	LASSO, PLS	-	-	-	2415	134.11749	LC/MS Pos Early	-	-
X - 12221	-	LASSO, PLS	-	-	-	1467	203.99752	LC/MS Neg	-	-
X - 12722	-	-	-	RF, LASSO, PLS	-	1871	249.00774	LC/MS Neg	-	-
X - 12726	-	-	-	LASSO, PLS	-	1942	233.01287	LC/MS Neg	-	-
X - 12823	-	-	-	RF, PLS	-	2815	167.0383	LC/MS Neg	-	-
X - 13844	-	-	RF, PLS	-	-	1442	209.05715	LC/MS Neg	-	-
X - 17010	-	LASSO, PLS	-	RF, PLS	-	3178	189.11359	LC/MS Neg	-	-
gentisic acid-5-glucoside	-	-	LASSO, PLS	-	-	1434	315.07215	LC/MS Neg	10914066	Xenobiotics
X - 23159 - retired for chenodeoxycholic acid sulfate (1)	-	LASSO, PLS	-	-	-	4548	235.11739	LC/MS Neg	-	-
X - 23587	-	LASSO, PLS	LASSO, PLS	-	-	1230	132.10181	LC/MS Pos Early	-	-
X - 23665	-	-	-	RF, PLS	-	2894	217.15428	LC/MS Pos Early	-	-

2-hydroxybutyrate/2-hydroxyisobutyrate	-	LASSO, PLS	-	-	-	1258	103.04006	LC/MS Polar	-	Amino Acid
X - 24330	-	-	-	LASSO, PLS	-	1546	225.07673	LC/MS Neg	-	-
X - 24333	-	-	RF, PLS	RF, LASSO, PLS	-	1789	202.10837	LC/MS Neg	-	-
X - 24413	-	-	-	LASSO, PLS	-	2795	159.14909	LC/MS Pos Early	-	-
X - 24435	-	LASSO, PLS	-	-	-	6625	465.30368	LC/MS Neg	-	-
X - 24470	-	-	-	LASSO, PLS	-	2252	306.15433	LC/MS Pos Early	-	-
X - 24543	-	-	LASSO, PLS	-	-	2823	275.02364	LC/MS Neg	-	-
3-hydroxyhexanoate	-	-	-	RF, PLS	-	1725	131.07136	LC/MS Neg	151492	Lipid
caffeic acid sulfate	-	LASSO, PLS	-	RF, LASSO, PLS	HMDB41708	1307	258.99179	LC/MS Polar	-	Xenobiotics
3-hydroxybutyrate (BHBA)	-	RF, LASSO, PLS	RF, PLS	RF, LASSO, PLS	HMDB00357	1444	103.04007	LC/MS Polar	441	Lipid
2-butenoylglycine	-	-	-	RF, PLS	-	1380	142.05096	LC/MS Neg	6303498	Lipid
3-heptenoylglutamine	-	LASSO, PLS	-	-	-	3220	255.13503	LC/MS Neg	-	Lipid
androsterone glucuronide	-	LASSO, PLS	-	-	HMDB02829	4953	465.24939	LC/MS Neg	114833	Lipid
3-carboxy-4-methyl-5-pentyl-2-furanpropionate (3-CMPFP)	-	LASSO, PLS	LASSO, PLS	-	-	4000	267.12379	LC/MS Neg	194501	Lipid
glucuronide of C10H18O2 (8)*	-	RF, PLS	RF, PLS	-	-	3865	345.1555	LC/MS Neg	-	PCM
glucuronide of C10H18O2 (9)*	-	RF, LASSO, PLS	RF, PLS	-	-	4010	345.1555	LC/MS Neg	-	PCM
glucuronide of C10H14O2 (2)*	-	-	RF, PLS	-	-	3154	341.12419	LC/MS Neg	-	PCM
glucuronide of C8H14O2 (6)*	-	-	-	LASSO, PLS	-	4027	317.12419	LC/MS Neg	-	PCM
glutamine conjugate of C8H12O4 (2)*	-	LASSO, PLS	LASSO, PLS	-	-	1143	299.12486	LC/MS Neg	-	PCM
gamma-CEHC taurine*	-	RF, PLS	RF, PLS	-	-	3917	370.13298	LC/MS Neg	-	Cofactors and Vitamins
4-hydroxycatechol sulfate	-	LASSO, PLS	-	-	-	1210	204.98123	LC/MS Neg	-	Xenobiotics
(2,4 or 2,5)-dimethylphenol sulfate	-	-	-	RF, LASSO, PLS	-	3474	201.0227	LC/MS Neg	-	Xenobiotics
1-carboxyethylisoleucine	-	-	-	RF, LASSO, PLS	-	2628	202.10848	LC/MS Neg	-	Amino Acid

4-acetylcatechol sulfate (1)	-	RF, LASSO, PLS	RF, LASSO, PLS	-	-	1774	230.99688	LC/MS Neg	-	Xenobiotics
4-acetylcatechol sulfate (2)	-	LASSO, PLS	-	-	-	2193	230.99688	LC/MS Neg	-	Xenobiotics
hydroxy-N6,N6,N6-trimethyllysine*	-	LASSO, PLS	-	-	-	2759	205.15468	LC/MS Pos Early	-	Amino Acid
N,N-dimethylalanine	-	RF, LASSO, PLS	RF, LASSO, PLS	-	-	1370	118.08626	LC/MS Pos Early	5488191	Amino Acid
pentose acid*	-	LASSO, PLS	-	-	-	2180	165.04046	LC/MS Polar	-	PCM

Metabolites with a prefix “X-” followed by a number (e.g., X - 17679) are molecular features whose biochemical identities are unknown, but their monoatomic mass and retention time (RT) were characterized. *Indicates metabolites that were not confirmed based on a standard, but Metabolon are confident in its identity.

Abbreviations: 2PYr, N1-Methyl-2-pyridone-5-carboxamide; AAMU, 5-acetylamino-6-amino-3-methyluracil; AS, added sugar; LASSO, Least Absolute Shrinkage and Selection Operator with bagging algorithm; LNCSB, low- and no-calorie sweetened beverages; PCM, Partially characterized molecules; PLS, partial least squares; RF, random forest; RI, mass spectral fragmentation and retention index; SBs, total sweetened beverages; SSB, sugar sweetened beverages.

Supplemental Table 3. Machine learning selections of food-related metabolites in 'young adult plasma' (*n* = 195)

metabolite	LNCSB	SSB	SBs	AS	HMDB ID	RI	Mass	Platform	Pubchem ID	Super pathway
citrate	RF, LASSO, PLS	-	-	-	HMDB00094	582	191.0197	LC/MS Neg	311	Energy
1-methylxanthine	RF, LASSO, PLS	RF, LASSO, PLS	RF, LASSO, PLS	RF, PLS	HMDB10738	1568.8	165.0418	LC/MS Neg	80220	Xenobiotics
aconitate [cis or trans]	RF, LASSO, PLS	-	-	-	-	580	173.0092	LC/MS Neg	-	Energy
adipoylcarnitine (C6-DC)	RF, LASSO, PLS	-	RF, PLS	-	HMDB61677	2570	290.1598	LC/MS Pos Early	71296139	Lipid
X - 24951	RF, LASSO, PLS	-	LASSO, PLS	-	-	-	-	LC/MS Neg	-	-
3,5-dichloro-2,6-dihydroxybenzoic acid	RF, LASSO, PLS	-	RF, LASSO, PLS	RF, LASSO, PLS	-	693	220.9414	LC/MS Polar	-	Xenobiotics
threonine	RF, PLS	-	-	-	HMDB00167	1514	120.0655	LC/MS Pos Early	6288	Amino Acid
choline	RF, PLS	-	-	-	HMDB00097	1961	104.107	LC/MS Pos Early	305	Lipid
5-hydroxylysine	RF, PLS	-	-	-	HMDB00450	2790	163.1077	LC/MS Pos Early	1029	Amino Acid
paraxanthine	RF, PLS	-	-	-	HMDB01860	2279	179.0575	LC/MS Neg	4687	Xenobiotics
theophylline	RF, PLS	-	-	-	HMDB01889	2356.9	179.0575	LC/MS Neg	2153	Xenobiotics
3-ureidopropionate	RF, PLS	-	-	-	HMDB00026	875	133.0608	LC/MS Pos Early	111	Nucleotide
1,3-dimethylurate	RF, PLS	LASSO, PLS	LASSO, PLS	-	HMDB01857	1671.4	195.0524	LC/MS Neg	70346	Xenobiotics
AAMU	RF, PLS	RF, LASSO, PLS	RF, LASSO, PLS	LASSO, PLS	HMDB04400	1710	197.068	LC/MS Polar	88299	Xenobiotics
hydroquinone sulfate	RF, PLS	-	LASSO, PLS	-	HMDB02434	1395	188.9863	LC/MS Neg	161220	Xenobiotics
octadecanedioate (C18)	RF, PLS	-	-	-	HMDB00782	5043	313.2384	LC/MS Neg	70095	Lipid
erythronate*	RF, PLS	-	-	-	HMDB00613	2186	135.0299	LC/MS Polar	2781043	Carbohydrate
6-oxopiperidine-2-carboxylate	RF, PLS	-	-	-	HMDB61705	965	142.051	LC/MS Neg	3014237	Amino Acid
2-piperidinone	RF, PLS	-	-	-	HMDB11749	1675	100.0757	LC/MS Pos Early	12665	Xenobiotics
methionine sulfone	RF, PLS	-	-	-	-	1250	182.0482	LC/MS Pos Early	69961	Amino Acid
X - 11381	LASSO, PLS	-	-	-	-	-	-	LC/MS Neg	-	-
X - 12847	RF, PLS	-	-	-	-	-	-	LC/MS Neg	-	-

methyl-4-hydroxybenzoate sulfate	RF, PLS	-	-	-	-	2873	230.9969	LC/MS Neg	-	Xenobiotics
3beta-hydroxy-5-cholestenoate	RF, PLS	-	-	LASSO, PLS	-	5398	415.3218	LC/MS Neg	165511	Lipid
gamma-glutamyl-alpha-lysine	RF, PLS	-	-	-	-	2784	276.1554	LC/MS Pos Early	65254	Peptide
caffeine	RF, PLS	LASSO, PLS	LASSO, PLS	-	HMDB01847	2106	195.0877	LC/MS Pos Early	2519	Xenobiotics
glutamate	RF, PLS	-	-	-	HMDB00148	1500	148.0604	LC/MS Pos Early	611	Amino Acid
histidine	RF, PLS	-	-	-	HMDB00177	755.9	154.0622	LC/MS Neg	6274	Amino Acid
3-hydroxyoleate*	RF, PLS	-	-	-	-	5508	297.2435	LC/MS Neg	-	Lipid
octadecenedioate (C18:1-DC)	LASSO, PLS	-	-	-	-	4875	311.2228	LC/MS Neg	-	Lipid
octadecanedioylcarnitine (C18-DC)*	LASSO, PLS	-	-	-	-	1227	458.3476	LC/MS Pos Late	-	Lipid
3-hydroxybutyroylglycine	RF, PLS	-	-	-	-	978	162.0761	LC/MS Pos Early	-	Lipid
2R,3R-dihydroxybutyrate	RF, PLS	-	-	-	HMDB00498	1543	119.035	LC/MS Polar	13120901	Lipid
glutamine conjugate of C6H10O2 (1)*	RF, PLS	-	LASSO, PLS	RF, PLS	-	2404	241.1194	LC/MS Neg	-	PCM
glutamine conjugate of C6H10O2 (2)*	RF, PLS	-	-	-	-	2526	241.1194	LC/MS Neg	-	PCM
3-bromo-5-chloro-2,6-dihydroxybenzoic acid*	LASSO, PLS	-	-	-	-	4505	264.8909	LC/MS Neg	-	Xenobiotics
ornithine	-	-	-	RF, PLS	HMDB03374	2800	133.0972	LC/MS Pos Early	6262	Amino Acid
gamma-glutamylphenylalanine	-	-	-	RF, PLS	HMDB00594	1825	293.1143	LC/MS Neg	111299	Peptide
gamma-glutamyltryptophan	-	-	-	LASSO, PLS	HMDB29160	1960	332.1252	LC/MS Neg	3989307	Peptide
AFMU	-	-	-	LASSO, PLS	HMDB11105	1424	225.0629	LC/MS Polar	108214	Xenobiotics
5alpha-androstan-3alpha,17beta-diol disulfate	-	-	-	RF, LASSO, PLS	-	4275	225.0697	LC/MS Neg	-	Lipid
4-cholesten-3-one	-	LASSO, PLS	-	-	HMDB00921	2520	385.3465	LC/MS Pos Late	91477	Lipid
isoleucylglycine	-	-	-	LASSO, PLS	HMDB28907	1992	187.1088	LC/MS Neg	342532	Peptide
pregnanediol-3-glucuronide	-	RF, PLS	-	-	HMDB10318	5145	495.2963	LC/MS Neg	123796	Lipid
bilirubin	-	-	-	RF, PLS	HMDB00054	1840	585.2708	LC/MS Pos Late	5280352	Cofactors and Vitamins

gamma-glutamylmethionine	-	-	-	RF, PLS	HMDB29155	2640	279.1009	LC/MS Pos Early	7009567	Peptide
pyroglutamine*	-	-	-	RF, LASSO, PLS	-	1900	129.0659	LC/MS Pos Early	134508	Amino Acid
X - 11308	-	-	LASSO, PLS	-	-	-	-	LC/MS Neg	-	-
X - 13866	-	LASSO, PLS	-	-	-	-	-	LC/MS Neg	-	-
X - 11530	-	-	-	RF, PLS	-	-	-	LC/MS Neg	-	-
X - 12462	-	RF, PLS	RF, PLS	-	-	-	-	LC/MS Pos Early	-	-
X - 15492	-	-	-	RF, PLS	-	-	-	LC/MS Neg	-	-
X - 21736	-	-	RF, PLS	-	-	-	-	LC/MS Neg	-	-
etiocholanolone glucuronide	-	-	-	LASSO, PLS	HMDB04484	4915	465.2494	LC/MS Neg	270605	Lipid
X - 11858	-	-	-	LASSO, PLS	-	-	-	LC/MS Neg	-	-
X - 13431	-	-	-	RF, PLS	-	-	-	LC/MS Pos Late	-	-
X - 17340	-	-	RF, PLS	-	-	-	-	LC/MS Neg	-	-
X - 16087	-	LASSO, PLS	LASSO, PLS	-	-	-	-	LC/MS Neg	-	-
N-formylphenylalanine	-	-	LASSO, PLS	-	-	2360	192.0666	LC/MS Neg	759256	Amino Acid
X - 23739	-	-	-	RF, LASSO, PLS	-	-	-	LC/MS Pos Early	-	-
alpha-ketobutyrate	-	-	-	LASSO, PLS	HMDB00005	940	101.0244	LC/MS Polar	58	Amino Acid
X - 24337	-	-	LASSO, PLS	-	-	-	-	LC/MS Neg	-	-
X - 24475	-	-	-	LASSO, PLS	-	-	-	LC/MS Pos Early	-	-
X - 24669	-	RF, LASSO, PLS	LASSO, PLS	LASSO, PLS	-	-	-	LC/MS Neg	-	-
X - 24849	-	-	-	RF, PLS	-	-	-	LC/MS Neg	-	-
arachidonoylcarnitine (C20:4)	-	RF, PLS	-	RF, PLS	-	1353	448.3422	LC/MS Pos Late	-	Lipid
dihomo-linolenoylcarnitine (C20:3n3 or 6)*	-	LASSO, PLS	RF, LASSO, PLS	RF, LASSO, PLS	-	1392	450.3578	LC/MS Pos Late	-	Lipid
carotene diol (1)	-	RF, LASSO, PLS	LASSO, PLS	RF, LASSO, PLS	-	1720	568.4276	LC/MS Pos Late	-	Cofactors and Vitamins
cortolone glucuronide (1)	-	-	-	LASSO, PLS	-	4600	541.2654	LC/MS Neg	-	Lipid
2-hydroxynervonate*	-	RF, PLS	RF, PLS	-	-	6345	381.3374	LC/MS Neg	5312783	Lipid

2-hydroxyphytanate*	-	RF, LASSO, PLS	-	-	-	5751	327.2905	LC/MS Neg	189026	Lipid
3-CMPFP	-	LASSO, PLS	LASSO, PLS	-	HMDB61643	4000	267.1238	LC/MS Neg	194501	Lipid
sulfate of piperine metabolite C18H21NO3 (3)*	-	LASSO, PLS	LASSO, PLS	LASSO, PLS	-	4630	378.1017	LC/MS Neg	-	Xenobiotics
linolenamide (18:3)*	-	-	-	RF, LASSO, PLS	-	1510	278.2479	LC/MS Pos Late	-	Lipid
palmitoyl-sphingosine-phosphoethanolamine (d18:1/16:0)	-	-	-	LASSO, PLS	-	2207	661.5279	LC/MS Pos Late	-	Lipid
cholesterol	-	LASSO, PLS	RF, PLS	RF, LASSO, PLS	HMDB00067	2707	369.3516	LC/MS Pos Late	11025495	Lipid
N,N-dimethylalanine	-	-	-	LASSO, PLS	-	1370	118.0863	LC/MS Pos Early	5488191	Amino Acid
branched-chain, straight-chain, or cyclopropyl 10:1 fatty acid (1)*	-	LASSO, PLS	-	-	-	4805	169.1234	LC/MS Neg	-	PCM
decadienedioic acid (C10:2-DC)	-	RF, PLS	-	-	-	2329.7	197.0819	LC/MS Polar	-	Lipid

Metabolites with a prefix “X-” followed by a number (e.g., X - 17679) are molecular features whose biochemical identities are unknown, but their monoatomic mass and retention time (RT) were characterized. *Indicates metabolites that were not confirmed based on a standard, but Metabolon are confident in its identity.

Abbreviations: 3-CMPFP, 3-carboxy-4-methyl-5-pentyl-2-furanpropionate; AAMU, 5-acetylamino-6-amino-3-methyluracil; AFMU, 5-acetylamino-6-formylamino-3-methyluracil; AS, added sugar; LASSO, Least Absolute Shrinkage and Selection Operator with bagging algorithm; LNCSB, low- and no-calorie sweetened beverages; PCM, partially characterized molecules; PLS, partial least squares; RF, random forest; RI, mass spectral fragmentation and retention index; SBs, total sweetened beverages; SSB, sugar sweetened beverages.

Supplemental Table 4. Metabolite features associated with SBs and AS intake in children (*n* = 297)

Foods	feature	Mass	RT (min)	m/z	Annotation[1]	MSI
SSB, SB	121.91727@0.601	121.91727	0.601	122.92455	—	—
SSB	128.0841@3.753	128.0841	3.753	127.0768	—	—
SSB	129.96719@0.626	129.96719	0.626	128.95991	—	—
SB	134.10968@4.712	134.10968	4.712	135.11696	—	—
SSB	136.03732@0.724	136.03732	0.724	135.03004	—	—
SB	142.02683@2.132	142.02683	2.132	143.0341	—	—
SSB	148.08901@3.889	148.08901	3.889	149.09629	—	—
AS	150.06827@4.068	150.06827	4.068	149.0610	—	—
SSB, SB	152.12024@4.714	152.12024	4.714	153.12752	—	—
AS	153.04277@2.289	153.04277	2.289	154.05005	—	—
AS	158.05809@2.816	158.05809	2.816	157.05081	—	—
AS	165.0652@1.186	165.0652	1.186	166.07248	7-Methylguanine (HMDB000089)	1
AS	165.07939@2.148	165.07939	2.148	166.08667	—	—
AS	166.04911@1.902	166.04911	1.902	167.05639	—	—
AS	168.02863@0.967	168.02863	0.967	167.02135	Uric acid (HMDB0000289)	2
LNCSB, SB	168.05358@1.786	168.05358	1.786	169.06086	—	—
SB	176.06858@3.179	176.06858	3.179	175.0613	—	—
AS	180.06467@2.415	180.06467	2.415	181.07195	Theobromine (HMDB0002825)	1
LNCSB	182.99887@2.451	182.99887	2.451	181.99159	Saccharin (HMDB0029723)	2
SSB	183.05335@1.813	183.05335	1.813	182.04607	4-Pyridoxic acid (HMDB0000017)	2
LNCSB, SB, AS	184.08475@1.608	184.08475	1.608	185.09203	-	
SB	189.04266@3.053	189.04266	3.053	190.04994	Kynurenic acid (HMDB0000715)	1
LNCSB	198.1007@2.172	198.1007	2.172	199.10798	—	—
AS	214.08427@3.876	214.08427	3.876	213.07699	—	—
SB	214.12043@5.044	214.12043	5.044	213.11315	—	—
SSB	220.00374@1.773	220.00374	1.773	221.01102	—	—
AS	228.11091@0.776	228.11091	0.776	227.10363	—	—
SB, AS	233.03563@1.471	233.03563	1.471	234.04291	—	—
AS	234.01955@2.357	234.01955	2.357	233.01227	—	—
AS	244.08815@2.104	244.08815	2.104	245.09543	—	—
SSB, AS	256.14227@3.906	256.14227	3.906	257.14955	—	—
AS	256.14245@3.937	256.14245	3.937	257.14973	—	—
AS	258.15795@4.241	258.15795	4.241	259.16523	—	—
AS	258.15815@4.273	258.15815	4.273	257.15087	—	—
SSB, SB	259.99915@3.019	259.99915	3.019	258.99187	—	—
SB	261.12113@0.936	261.12113	0.936	262.12841	—	—
AS	280.10589@2.888	280.10589	2.888	281.11317	Aspartylphenylalanine (HMDB0000706)	2
SSB, SB	282.11048@4.718	282.11048	4.718	281.1032	—	—
AS	284.0897@3.588	284.0897	3.588	283.08242	—	—
AS	290.09368@2.095	290.09368	2.095	289.0864	—	—
AS	301.11611@3.557	301.11611	3.557	302.12339	—	—
SSB, SB, AS	311.20953@4.339	311.20953	4.339	312.21681	Decadienoylcarnitine (Acylcarnitine C10:2)	2
AS	342.1315@4.916	342.1315	4.916	341.12422	—	—
SSB, SB	345.21495@2.18	345.21495	2.18	346.22223	—	—
SSB, SB, AS	346.16262@3.85	346.16262	3.85	345.15534	—	—
AS	346.16262@4.744	346.16262	4.744	345.15534	—	—
SSB	346.16267@4.709	346.16267	4.709	347.16995	—	—
SSB, SB	364.09779@0.732	364.09779	0.732	365.10507	—	—
SSB, SB, AS	378.0928@0.759	378.0928	0.759	377.08552	—	—
AS	440.08768@0.759	440.08768	0.759	439.0804	—	—
SSB	453.19967@4.707	453.19967	4.707	454.20695	—	—

[1]Annotations with a dash (—) means biochemical identity is unknown but its monoatomic mass, retention time (RT), and mass-to-charge ratio (m/z) were characterized.

Only metabolites with FDR-adjusted q-value < 0.05 are shown, from models adjusted for age, sex, and energy intake, with a random intercept for each participant. Food-specific metabolites as well as non-specific metabolites (associated with multiple food groups) as shown under "foods". Abbreviations: AS, added sugar; LNCSB, low- and no-calorie sweetened beverages; MSI, Metabolites standards initiative; SBs, total sweetened beverages; SSB, sugar sweetened beverages.

Supplemental Table 5: Metabolites associated with SBs and AS intake in 'adolescent urine' (*n* = 339)

Foods	Metabolite	Mass	RT	HMDB ID	Super pathway
AS	sucrose	341.10894	865	HMDB00258	Carbohydrate
SSB, SB	3-hydroxyisobutyrate	103.04007	1619	HMDB00336	Amino Acid
SSB	3-hydroxypropanoate	89.02442	1845	HMDB00700	Lipid
AS	indolelactate	204.06661	2286	HMDB00671	Amino Acid
LNCSB	1,6-anhydroglucose	207.05102	1175.5	HMDB00640	Xenobiotics
LNCSB, SB	saccharin	181.99173	2000	HMDB29723	Xenobiotics
SSB, SB	2-hydroxyoctanoate	159.10266	3736.8	HMDB02264	Lipid
AS	phenyllactate (PLA)	165.05571	908	HMDB00779	Amino Acid
SSB, SB	N-formylmethionine	176.03869	1543.8	HMDB01015	Amino Acid
AS	1-methylhistidine	168.07785	1227	HMDB00001	Amino Acid
SSB	3-hydroxysebacate	217.10814	2411	HMDB00350	Lipid
AS	3-hydroxyanthranilate	154.04987	2407	HMDB01476	Amino Acid
AS	decanoylcarnitine (C10)	316.24824	1130	HMDB00651	Lipid
SSB, AS	1-methylxanthine	165.0418	1568.8	HMDB10738	Xenobiotics
SSB, SB	5-acetylamino-6-amino-3-methyluracil	197.06801	1710	HMDB04400	Xenobiotics
SSB, SB	hydroquinone sulfate	188.98631	1395	HMDB02434	Xenobiotics
SSB, SB	3-methyladipate	159.06628	2865	HMDB00555	Lipid
SSB	N-methylhydantoin	113.03565	1425	HMDB03646	Amino Acid
SSB	carboxyethyl-GABA	176.09174	2085	HMDB02201	Amino Acid
SSB, AS	cis-urocanate	137.03565	1200	HMDB34174	Amino Acid
SSB, SB	2-oxo-1-pyrrolidinepropionate	158.08117	1600		Xenobiotics
SSB, SB	N1-Methyl-2-pyridone-5-carboxamide	151.0513	1668	HMDB04193	Cofactors and Vitamins
LNCSB, AS	benzoylcarnitine*	266.13869	3041		Xenobiotics
SSB	2-aminophenol sulfate	188.0023	1677	HMDB61116	Xenobiotics
SB	gamma-CEHC	263.12888	3843	HMDB01931	Cofactors and Vitamins
SSB, SB	X - 21258	213.02316	3915		
SSB, SB	X - 21312	243.03368	2757.7		
AS	X - 11478	165.09214	4285		
AS	X - 12472	241.11935	2528		
SB	X - 21825	234.07805	3220		
SB	X - 21831	363.16759	3480		
AS	X - 21847	403.10512	4270		
AS	X - 18887	328.15182	2229		
AS	X - 18410	305.07041	1538		
LNCSB	X - 11612	283.06849	1633		
AS	X - 11640	377.07052	3769		
AS	X - 12101	164.07386	2700		
AS	X - 11858	437.0555	4414		
SSB, SB	X - 19299	227.12896	3728		
SSB, SB	X - 19497	227.12921	3643		
AS	X - 17327	257.14918	2750		
SSB, SB	X - 17328	308.18508	910		
SSB	X - 18126	134.11749	2415		
SSB	X - 12221	203.99752	1467		

AS	X - 12722	249.00774	1871		
AS	X - 12726	233.01287	1942		
AS	X - 12823	167.0383	2815		
SB	X - 13844	209.05715	1442		
SSB, AS	X - 17010	189.11359	3178		
LNCSB	X - 17349	367.10412	2497		
LNCSB, SSB, SB, AS	X - 17679	199.09797	1775		
LNCSB, SB	acesulfame	161.98665	1581	HMDB33585	Xenobiotics
SB	gentisic acid-5-glucoside	315.07215	1434		Xenobiotics
SSB, SB	X - 23587	132.10181	1230		
AS	X - 24330	225.07673	1546		
SB, AS	X - 24333	202.10837	1789		
AS	X - 24413	159.14909	2795		
LNCSB	X - 24414	234.08027	1965		
SB	X - 24543	275.02364	2823		
AS	3-hydroxyhexanoate	131.07136	1725		Lipid
SSB, AS	caffeic acid sulfate	258.99179	1307	HMDB41708	Xenobiotics
SSB, SB, AS	3-hydroxybutyrate (BHBA)	103.04007	1444	HMDB00357	Lipid
LNCSB, SB	X - 24794	123.05621	1131		
SSB	3-heptenoylglutamine	255.13503	3220		Lipid
SSB, SB	3-carboxy-4-methyl-5-pentyl-2-furanpropionate (3-CMPFP)	267.12379	4000		Lipid
SSB, SB	glucuronide of C10H18O2 (8)*	345.1555	3865		PCM
SB	glucuronide of C10H18O2 (9)*	345.1555	4010		PCM
SB	glucuronide of C10H14O2 (2)*	341.12419	3154		PCM
AS	glucuronide of C8H14O2 (6)*	317.12419	4027		PCM
SSB, SB	glutamine conjugate of C8H12O4 (2)*	299.12486	1143		PCM
SB	gamma-CEHC taurine*	370.13298	3917		Cofactors and Vitamins
SSB	4-hydroxycatechol sulfate	204.98123	1210		Xenobiotics
AS	(2,4 or 2,5)-dimethylphenol sulfate	201.0227	3474		Xenobiotics
AS	1-carboxyethylisoleucine	202.10848	2628		Amino Acid
SSB, SB	4-acetylcatechol sulfate (1)	230.99688	1774		Xenobiotics
SSB	4-acetylcatechol sulfate (2)	230.99688	2193		Xenobiotics
SSB	hydroxy-N6,N6,N6-trimethyllysine*	205.15468	2759		Amino Acid
SSB, SB	N,N-dimethylalanine	118.08626	1370		Amino Acid
LNCSB	3-hydroxyisonicotinic acid	138.01966	1497		Xenobiotics
LNCSB	5-hydroxy-2-methylpyridine sulfate	188.0023	2074		Xenobiotics
SSB	pentose acid*	165.04046	2180		PCM

The food-related metabolites summarized in this table had FDR-adjusted q-value < 0.05, from models adjusted for age, sex, energy intake, physical activity, alcohol and smoking status. Food-specific metabolites as well as non-specific metabolites are shown under “Foods”.

Metabolites with a prefix “X-” followed by a number (e.g., X - 17679) are molecular features whose biochemical identities are unknown, but their monoatomic mass and retention time (RT) were characterized. *Indicates metabolites that were not confirmed based on a standard, but Metabolon are confident in its identity.

Abbreviations: AS, added sugar; LNCSB, low- and no-calorie sweetened beverages; PCM, partially characterized molecules; SBs, total sweetened beverages; SSB, sugar sweetened beverages.

Supplemental Table 6. Metabolites associated with SBs and AS intake in 'young adult plasma' (*n* = 195)

Foods	Metabolite	Mass	RT	HMDB	Super pathway
SSB, SB	1,3-dimethylurate	195.0524	1671.4	HMDB01857	Xenobiotics
SSB, SB, AS	1-methylxanthine	165.0418	1568.8	HMDB10738	Xenobiotics
SSB, SB, AS	AAMU	197.068	1710	HMDB04400	Xenobiotics
SB	hydroquinone sulfate	188.9863	1395	HMDB02434	Xenobiotics
SSB	4-cholesten-3-one	385.3465	2520	HMDB00921	Lipid
SB	X – 11308	-	-	-	-
SSB	X – 13866	-	-	-	-
AS	etiocholanolone glucuronide	465.2494	4915	HMDB04484	Lipid
SB	X - 17340	-	-	-	-
SSB, SB	X - 16087	-	-	-	-
SB	N-formylphenylalanine	192.0666	2360	-	Amino Acid
SB	X - 24337	-	-	-	-
LNCSB, SB	adipoylcarnitine (C6-DC)	290.1598	2570	HMDB61677	Lipid
SSB	X – 24669	-	-	-	-
SSB, SB	caffeine	195.0877	2106	HMDB01847	Xenobiotics
SB	dihomo-linolenoylcarnitine (C20:3n3 or 6)*	450.3578	1392	-	Lipid
SSB	carotene diol (1)	568.4276	1720	-	Cofactors and Vitamins
SB	X - 24951			-	-
LNCSB	octadecanedioylcarnitine (C18-DC)*	458.3476	1227	-	Lipid
SSB, SB	3-CMPFP	267.1238	4000	HMDB61643	Lipid
SSB	branched-chain, straight-chain, or cyclopropyl 10:1 fatty acid (1)*	169.1234	4805	-	PCM
SB	glutamine conjugate of C6H10O2 (1)*	241.1194	2404	-	PCM
LNCSB	3-bromo-5-chloro-2,6-dihydroxybenzoic acid*	264.8909	4505	-	Xenobiotics

The food-related metabolites summarized in this table had FDR-adjusted q-value < 0.05. All models were adjusted for age, sex, energy intake, physical activity, alcohol and smoking status, number of dietary assessments, and the difference in time between dietary assessment and blood draw. Food-specific metabolites as well as non-specific metabolites (associated with multiple food groups) as shown under "Foods".

Metabolites with a prefix "X-" followed by a number (e.g., X – 11308) are molecular features whose biochemical identities could not be identified, but their monoatomic mass and retention time (RT) were characterized. *Indicates metabolites that were not confirmed based on a standard, but Metabolon are confident in its identity.

Abbreviations: 3-CMPFP, 3-carboxy-4-methyl-5-pentyl-2-furanpropionate; AAMU, 5-acetylamino-6-amino-3-methyluracil; AS, added sugar; LNCSB, low- and no-calorie sweetened beverages; SB, sweetened beverages; SSB, sugar sweetened beverages.

Supplemental Table 7: Associations of coffee intake with caffeine metabolites in 'adolescent urine' (n = 339) and 'young adult plasma' (n = 195)

**Adolescent Urine**				**95% CI**	
**Metabolite**	**HMDB ID**	**Super pathway**	**Estimate**	**Lower**	**Upper**
1-methylurate	HMDB03099	Xenobiotics	0.0048	0.0037	0.0059
AAMU	HMDB04400	Xenobiotics	0.0046	0.0035	0.0057
1-methylxanthine	HMDB10738	Xenobiotics	0.0047	0.0035	0.0058
paraxanthine	HMDB01860	Xenobiotics	0.0045	0.0034	0.0056
theophylline	HMDB01889	Xenobiotics	0.0044	0.0033	0.0055
caffeine	HMDB01847	Xenobiotics	0.0039	0.0028	0.0050
1,3,7-trimethylurate	HMDB02123	Xenobiotics	0.0038	0.0027	0.0049
1,7-dimethylurate	HMDB11103	Xenobiotics	0.0038	0.0027	0.0049
7-methylurate	HMDB11107	Xenobiotics	0.0005	-0.0007	0.0017
7-methylxanthine	HMDB01991	Xenobiotics	0.0003	-0.0009	0.0015
theobromine	HMDB02825	Xenobiotics	-0.0003	-0.0015	0.0009
3-methylurate*		Xenobiotics	0.0001	-0.0011	0.0013
3,7-dimethylurate	HMDB01982	Xenobiotics	-0.0001	-0.0012	0.0011
3-methylxanthine	HMDB01886	Xenobiotics	-0.0001	-0.0013	0.0012
**Young Adult Plasma**					
AAMU	HMDB04400	Xenobiotics	0.0064	0.0037	0.0092
theophylline	HMDB01889	Xenobiotics	0.0055	0.0027	0.0084
1,3-dimethylurate	HMDB01857	Xenobiotics	0.0055	0.0027	0.0084
1-methylxanthine	HMDB10738	Xenobiotics	0.0052	0.0024	0.0081
1,7-dimethylurate	HMDB11103	Xenobiotics	0.0050	0.0022	0.0079
AFMU	HMDB11105	Xenobiotics	0.0049	0.0020	0.0078
paraxanthine	HMDB01860	Xenobiotics	0.0049	0.0020	0.0078
caffeine	HMDB01847	Xenobiotics	0.0037	0.0008	0.0066
7-methylxanthine	HMDB01991	Xenobiotics	0.0014	-0.0017	0.0044
theobromine	HMDB02825	Xenobiotics	0.0010	-0.0019	0.0040
3-methylxanthine	HMDB01886	Xenobiotics	0.0009	-0.0021	0.0038
3,7-dimethylurate	HMDB01982	Xenobiotics	0.0003	-0.0027	0.0033

All caffeine and caffeine related metabolites measured in our data were analyzed.
Adolescent urine: Models were adjusted for age, sex, energy intake, physical activity, smoking and alcohol status.
Young adult plasma: All adjustments for adolescent urine, plus time difference between dietary assessment and blood draw (difference = age at blood draw – mean age of dietary assessments) and the number of dietary assessments to reflect its analytic design.
*Indicates metabolites that were not confirmed based on a standard, but Metabolon are confident in its identity.

Abbreviations: AAMU, 5-acetylamino-6-amino-3-methyluracil; AFMU, 5-acetylamino-6-formylamino-3-methyluracil; CI, confidence intervals;

Supplemental Table 8: Associations of SSB intake with all caffeine metabolites in 'adolescent urine' (n = 339) and 'young adult plasma' (n = 195)

**Adolescent Urine**				**95% CI**	
**Metabolite**	**HMDB ID**	**Super pathway**	**Estimate**	**Lower**	**Upper**
1-methylxanthine	HMDB10738	Xenobiotics	0.00054	0.00029	0.00078
AAMU	HMDB04400	Xenobiotics	0.00050	0.00026	0.00074
paraxanthine	HMDB01860	Xenobiotics	0.00047	0.00022	0.00072
theophylline	HMDB01889	Xenobiotics	0.00046	0.00022	0.00070
1,7-dimethylurate	HMDB11103	Xenobiotics	0.00044	0.00020	0.00069
1,3,7-trimethylurate	HMDB02123	Xenobiotics	0.00042	0.00018	0.00066
1-methylurate	HMDB03099	Xenobiotics	0.00040	0.00016	0.00065
caffeine	HMDB01847	Xenobiotics	0.00040	0.00016	0.00064
theobromine	HMDB02825	Xenobiotics	0.00006	-0.00019	0.00032
3-methylxanthine	HMDB01886	Xenobiotics	0.00006	-0.00019	0.00031
7-methylurate	HMDB11107	Xenobiotics	0.00004	-0.00022	0.00029
7-methylxanthine	HMDB01991	Xenobiotics	0.00004	-0.00022	0.00029
3,7-dimethylurate	HMDB01982	Xenobiotics	0.00003	-0.00021	0.00028
3-methylurate*		Xenobiotics	-0.00001	-0.00026	0.00024
**Young Adult Plasma**					
1-methylxanthine	HMDB10738	Xenobiotics	0.00104	0.00052	0.00156
1,7-dimethylurate	HMDB11103	Xenobiotics	0.00103	0.00051	0.00156
theophylline	HMDB01889	Xenobiotics	0.00101	0.00049	0.00154
caffeine	HMDB01847	Xenobiotics	0.00103	0.00049	0.00158
paraxanthine	HMDB01860	Xenobiotics	0.00101	0.00048	0.00155
AAMU	HMDB04400	Xenobiotics	0.00096	0.00045	0.00147
1,3-dimethylurate	HMDB01857	Xenobiotics	0.00091	0.00039	0.00144
AFMU	HMDB11105	Xenobiotics	0.00070	0.00015	0.00124
3,7-dimethylurate	HMDB01982	Xenobiotics	-0.00028	-0.00084	0.00028
7-methylxanthine	HMDB01991	Xenobiotics	-0.00007	-0.00064	0.00049
theobromine	HMDB02825	Xenobiotics	0.00007	-0.00048	0.00061
3-methylxanthine	HMDB01886	Xenobiotics	-0.00002	-0.00056	0.00053

All caffeine and caffeine related metabolites measured in our data were analyzed.
Adolescent urine: Models were adjusted for age, sex, energy intake, physical activity, smoking and alcohol status, chocolate, powdered instant beverages, and coffee intake.
Young adult plasma: Models includes all adjustments for adolescent urine samples, plus time difference between dietary assessment and blood draw (difference = age at blood draw – mean age of dietary assessments) and the number of dietary assessments to reflect its analytic design.
*Indicates metabolites that were not confirmed based on a standard, but Metabolon are confident in its identity based on their biochemical identification criteria and chemical properties.

Abbreviations: AAMU, 5-acetylamino-6-amino-3-methyluracil; AFMU, 5-acetylamino-6-formylamino-3-methyluracil; CI, confidence intervals;

Supplemental Figure 3. Assessing potential bias due to different imputation methods on food-metabolite associations

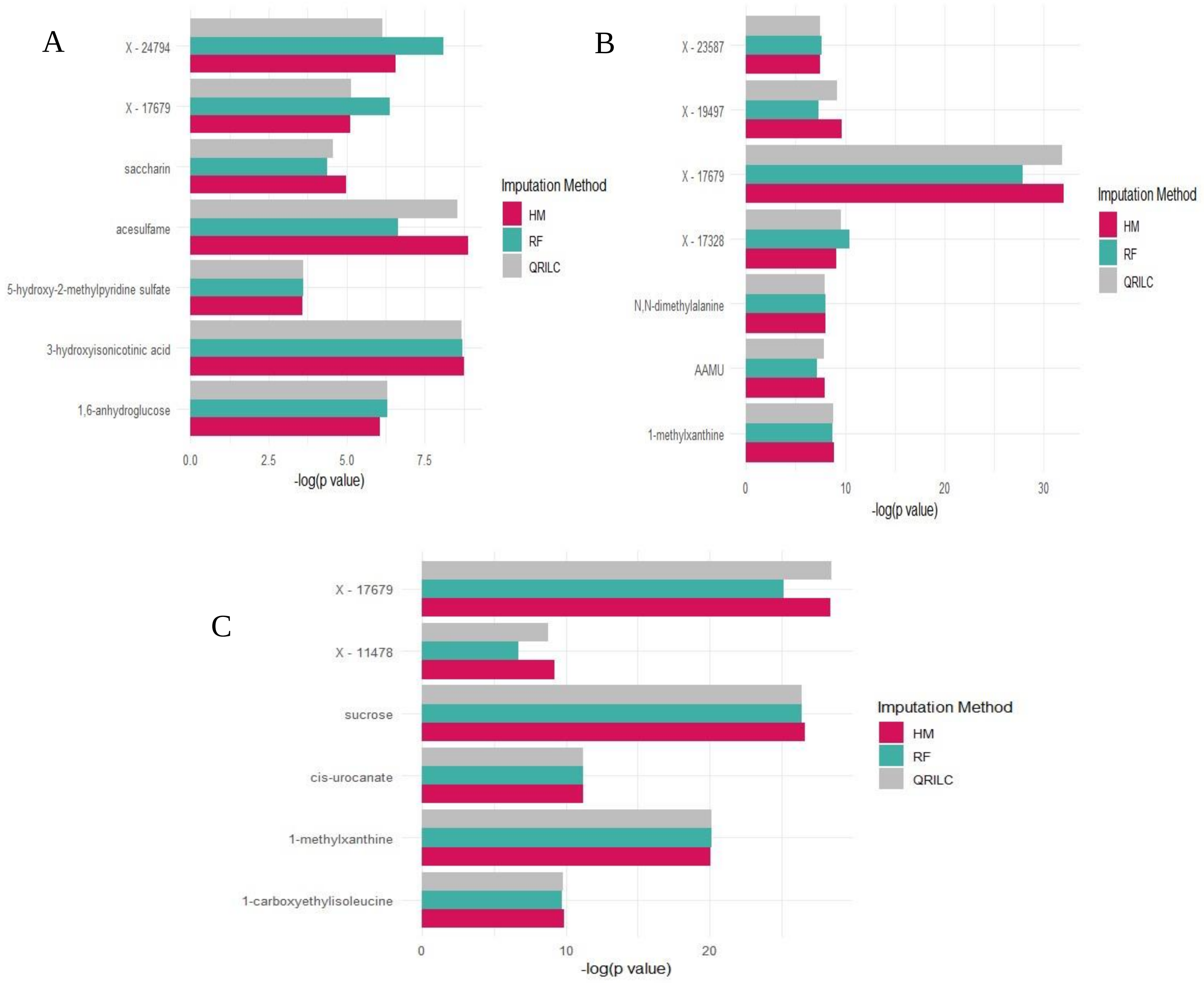

Supplemental Figure 3. Assessing potential bias due to different imputation methods on food-metabolite associations in 'adolescent urine', *n* = 339**. A** – LNCSB, low – and no-calorie sweetened beverages**; B** – SSB, Sugar-sweetened Beverages; **C** – AS, Added sugar models. Using adolescent urine analytic sample, we compared food-metabolite results (Half-minimum [HM] imputation) with other imputation methods for the assumption of left-censored data (Quantile regression imputation for left censored data, QRILC) and missing completely at random/missing at random (Random forest, RF). The results suggested that the imputation methods HM, QRILC, and RF yielded comparable results for most of the associations supporting our main findings. For visualization purposes, only the top metabolites (by regression estimate) with FDR-corrected p-value, q < 0.05 are shown. The models for food-metabolite associations were adjusted for age, sex, energy intake, physical activity, smoking, and alcohol status.

Supplemental Table 9. Food-related metabolites associated with adiposity using regularized regression in 'adolescent urine'

**metabolite**	**BMI** (*n* = 339)[1]	**% BF** (*n* = 339)[1]	**WC** (*n* = 231)[1]
decanoylcarnitine (C10)	0.23	0.19	0.30
acesulfame	0.18	0.10	0.11
3-hydroxyhexanoate	-0.14	-0.08	-0.2
X - 24333	0.14	—	—
gamma-CEHC taurine*	-0.13	-0.08	-0.11
X - 18887	-0.12	-0.04	-0.08
glutamine conjugate of C8H12O4 (2)*	-0.12	-0.03	-0.11
3-hydroxyisonicotinic acid	-0.10	-0.01	-0.11
N1-Methyl-2-pyridone-5-carboxamide	0.10	0.08	0.12
glucuronide of C8H14O2 (6)*	0.10	0.07	0.12
X - 17010	-0.09	-0.05	-0.08
carboxyethyl-GABA	-0.08	-0.07	-0.09
X - 11858	0.07	0.03	0.06
N,N-dimethylalanine	-0.07	-0.11	-0.12
X - 17679	-0.07	—	-0.13
3-CMPFP	0.06	0.04	0.03
X - 21847	0.06	—	0.03
3-methyladipate	-0.06	—	-0.02
phenyllactate (PLA)	0.05	—	—
X - 12472	0.05	—	0.07
cis-urocanate	-0.05	—	-0.14
X - 17328	0.05	—	—
X - 13844	-0.04	-0.09	-0.05
3-heptenoylglutamine	-0.04	—	—
3-hydroxysebacate	-0.04	-0.02	-0.05
1-carboxyethylisoleucine	0.03	—	0.11
pentose acid*	-0.03	—	—
X - 11612	-0.02	-0.03	-0.03
hydroxy-N6,N6,N6-trimethyllysine*	0.02	—	0.04
X - 24330	0.01	0.01	—
3-hydroxybutyrate (BHBA)	-0.01	—	—
3-hydroxypropanoate	0.01	0.05	0.04
X - 21258	—	-0.03	—
2-oxo-1-pyrrolidinepropionate	—	-0.01	—
caffeic acid sulfate	—	-0.01	-0.08
hydroquinone sulfate	—	-0.01	-0.03
saccharin	—	—	0.06
X - 24414	—	—	0.05
X - 21312	—	—	0.04
X - 12726	—	—	0.04
X - 21831	—	—	-0.03
2-hydroxyoctanoate	—	—	-0.01
3-hydroxyanthranilate	—	—	0.01

Linear regression models were applied to adjust metabolite levels for confounders (age, sex, energy intake, birthweight, time difference between urine collection and anthropometric measurements, physical activity, smoking, and alcohol status). For each of the adiposity measure (outcome variables), the confounder-adjusted metabolites (predictors) were then fit in an adaptive elastic-net regression model, using the inverse of the absolute ridge regression weights as penalty factors. We used the 'nestedcv' R package for nested cross-validation, training models in the inner loop and evaluating them in the outer loop as per author recommendations [4] .

Only food-related metabolites associated (non-zero values) with at least one of the three anthropometric measures are shown in this table.

[1]A dash [—] indicates that the metabolite had no association with that anthropometric measure.

Metabolites with a prefix 'X-' followed by a number (e.g., X - 24333) are molecular features whose biochemical identities could not be identified. *Indicates metabolites that were not confirmed based on a standard, but Metabolon Inc are confident in its identity based on their biochemical identification criteria and chemical properties.

Abbreviations: %BF, body fat percentage; 3-CMPFP, 3-carboxy-4-methyl-5-pentyl-2-furanpropionate; BMI, body mass index; WC, waist circumference.

Supplemental Table 10. Food-related metabolites associated with adiposity using regularized regression 'young adult plasma' (n = 195)

Metabolite	BMI[1]	% BF[1]	WC[1]
X - 17340	0.25	0.16	0.16
X - 24337	0.17	0.02	0.12
carotene diol (1)	-0.13	-0.07	-0.16
X - 13866	-0.13	-0.05	-0.09
X - 24669	-0.12	-0.08	-0.04
octadecanedioylcarnitine (C18-DC)*	-0.10	-0.10	-0.12
X - 11308	-0.09	-0.07	-0.07
5-acetylamino-6-amino-3-methyluracil	0.08	0.03	—
4-cholesten-3-one	0.07	0.05	—
adipoylcarnitine (C6-DC)	0.04	—	—
dihomo-linolenoylcarnitine (C20:3n3 or 6)*	-0.03	—	—
cyclopropyl 10:1 fatty acid (1)*	-0.02	-0.06	—
3-bromo-5-chloro-2,6-dihydroxybenzoic acid*	-0.01	-0.07	-0.02
X - 16087	—	0.02	0.03
N-formylphenylalanine	—	0.02	0.01
X - 24951	—	—	0.06
3-CMPFP	—	—	0.05
glutamine conjugate of C6H10O2 (1)*	—	—	-0.01

Linear regression models were applied to adjust metabolite levels for confounders (age, sex, energy intake, birthweight, time difference between blood collection and anthropometric measurements, physical activity, smoking, and alcohol status, time difference between dietary assessment and blood draw and the number of dietary assessments). For each of the adiposity measures (outcome variables), the confounder-adjusted metabolites (predictors) were then fit in an adaptive elastic-net regression model, using the inverse of the absolute ridge regression weights as penalty factors. We used the 'nestedcv' R package for nested cross-validation, training models in the inner loop and evaluating them in the outer loop as per author recommendations [4].

Only food-related metabolites associated (non-zero values) with at least one of the three anthropometric measures are shown in this table.

[1]A dash [—] indicates that the metabolite had no association with that anthropometric measure.

Metabolites with a prefix 'X-' followed by a number (e.g., X - 24333) are molecular features whose biochemical identities could not be identified. *Indicates metabolites that were not confirmed based on a standard, but Metabolon Inc are confident in its identity based on their biochemical identification criteria and chemical properties.

Abbreviations: % BF, body fat percentage; 3-CMPFP, 3-carboxy-4-methyl-5-pentyl-2-furanpropionate; BMI, body mass index; WC, waist circumference.

References

1. Wishart, D.S., Feunang, Y.D., Marcu, A., Guo, A.C., Liang, K., Vázquez-Fresno, R., Sajed, T., Johnson, D., Li, C., Karu, N., Sayeeda, Z., Lo, E., Assempour, N., Berjanskii, M., Singhal, S., Arndt, D., Liang, Y., Badran, H., Grant, J., Serra-Cayuela, A., Liu, Y., Mandal, R., Neveu, V., Pon, A., Knox, C., Wilson, M., Manach, C., Scalbert, A.: HMDB 4.0: the human metabolome database for 2018. Nucleic acids research (2018). https://doi.org/10.1093/nar/gkx1089
2. Smith, C.A., O'Maille, G., Want, E.J., Qin, C., Trauger, S.A., Brandon, T.R., Custodio, D.E., Abagyan, R., Siuzdak, G.: METLIN: a metabolite mass spectral database. Therapeutic drug monitoring (2005). https://doi.org/10.1097/01.ftd.0000179845.53213.39
3. Sumner, L.W., Amberg, A., Barrett, D., Beale, M.H., Beger, R., Daykin, C.A., Fan, T.W.-M., Fiehn, O., Goodacre, R., Griffin, J.L., Hankemeier, T., Hardy, N., Harnly, J., Higashi, R., Kopka, J., Lane, A.N., Lindon, J.C., Marriott, P., Nicholls, A.W., Reily, M.D., Thaden, J.J., Viant, M.R.: Proposed minimum reporting standards for chemical analysis Chemical Analysis Working Group (CAWG) Metabolomics Standards Initiative (MSI). Metabolomics : Official journal of the Metabolomic Society (2007). https://doi.org/10.1007/s11306-007-0082-2
4. Lewis M.J, Spiliopoulou A., Goldmann, K., Pitzalis, C., McKeigue, P., Barnes, M.R. nestedcv: An R package for fast implementation of nested cross-validation with embedded feature selection designed for transcriptomics and high-dimensional data. Bioinform Adv. 3 (2023), vbad048.
